# Tailor made magnetic nanolights: fabrication to cancer theranostics applications

**DOI:** 10.1039/d1na00447f

**Published:** 2021-10-25

**Authors:** Poushali Das, Sayan Ganguly, Shlomo Margel, Aharon Gedanken

**Affiliations:** Bar-Ilan Institute for Nanotechnology and Advanced Materials (BINA), Bar-Ilan University Ramat-Gan 5290002 Israel Aharon.Gedanken@biu.ac.il; Departments of Chemistry, Bar-Ilan University Ramat-Gan 5290002 Israel

## Abstract

Nanoparticles having magnetic and fluorescent properties could be considered as a gift to materials scientists due to their unique magneto-optical qualities. Multiple component particles can overcome challenges related with a single component and unveil bifunctional/multifunctional features that can enlarge their applications in diagnostic imaging agents and therapeutic delivery vehicles. Bifunctional nanoparticles that have both luminescent and magnetic features are termed as magnetic nanolights. Herein, we present recent progress of magneto-fluorescent nanoparticles (quantum dots based magnetic nanoparticles, Janus particles, and heterocrystalline fluorescent magnetic materials), comprehensively describing fabrication strategies, types, and biomedical applications. In this review, our aim is not only to encompass the preparation strategies of these special types of magneto-fluorescent nanomaterials but also their extensive applications in bioimaging techniques, cancer therapy (targeted and hyperthermic), and sustained release of active agents (drugs, proteins, antibodies, hormones, enzymes, growth factors).

## Abstract 2

This review covers the recent progress of magnetic nanolights, fabrication strategies, and application in cancer theranostics.

## Graphical abstract

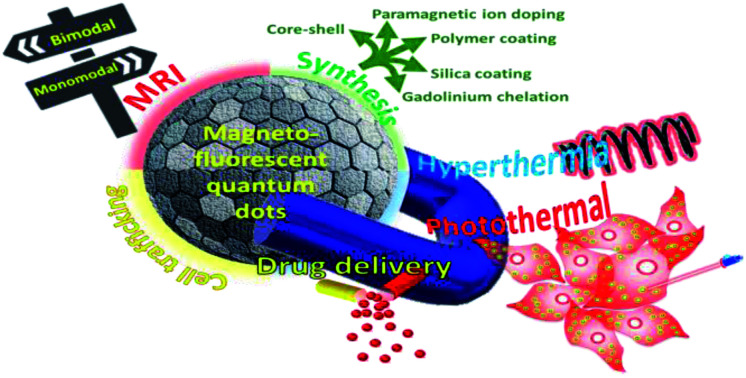

## 1. Introduction

Nanotechnology is a rapidly developing area, involving the fabrication and usage of nano-scaled materials and devices. Due to the innovations in nanotechnology, it was possible to incorporate various nanoparticles in one single hybrid nanostructure. Recently, different nanoparticles have shown promising features and superiority in the biomedical field. Among them, magnetic nanoparticles (MNPs), quantum dots (QDs), and the functional materials based on the two properties are the ones with the greatest applicative values.^1–3^ The amalgamation of the fluorescent and magnetic features in one entity opens up unique magneto-fluorescent multimodal nanomaterial for medical diagnostics and cancer therapy.^4,5^

As the focus of this discussed is solely on the biomedical area, the diagnostics, and therapies both have demand in the clinical world. Intricate problem diagnosis *via* multimodal imaging of organs with high precision manner is desired for physicians for better understanding. It is also important for medical researchers.^6,7^ ‘Single particle-multimodality’ is the key term adopted by the scientific communities where nanostructures are tailor-made for multimodal imaging.^8,9^ Very recently, combinational approaches have been taken for obtaining synergistic results of bio-imaging. As example, positron emission tomography (PET) and computer tomography (CT)^10^ are combined to deliver better biomedical detailing compared to single imaging techniques. PET and MRI are also used in several versatile commercial platforms as diagnostic tool. Besides these, optical imaging tools like fluorescence imaging and two-photon imaging are also drawing attention due to their relative low cost and of easy handling.^11,12^ Now a day these tools are mostly used to diagnose cancer in human organs. Cancer is the foremost cause of death worldwide. By 2030, 13.2 million deaths across the globe are forecasted by the United Nations.^13^ Cancer theranostics, *i.e.*, the combination of bioimaging diagnostics and cancer therapy has been developing toward personalized cancer treatment for the sake of patients by the past few decades.^14,15^ Advances in nanotechnology offered several benefits to the nanoscale systems (such as silica nanoparticles,^16–18^ carbon quantum dots,^19–24^ and iron oxide^25–28^) which had a significant impact on therapeutic, imaging systems, tissue engineering, and regenerative medicine.^29,30^ The combination of magnetic and luminescent properties in one nanocomposite could offer important biological uses for cancer therapy in different imaging methods such as *in vitro* and *in vivo* bioimaging including MRI, fluorescence bioimaging, and various therapeutic approaches (medical diagnostics, photodynamic therapy, magnetic hyperthermia and drug delivery).^31–33^ Therefore, magneto-fluorescent materials have been utilized in clinical applications as a multimodal diagnostic and therapeutic tool to identify, diagnose and simultaneously treat cancer diseases. Several researchers including our group has been working on the synthesis and applications of carbon dots and carbon dots conjugated systems since 2013, and are also involved in the development of magnetic nanoparticles and magneto-fluorescent nanoparticles for around 40 years.^34–41^ It has been seventeen years since Pankhurst *et al.* wrote their well-known review on magnetic nanoparticles in biomedicine.^42^ In the last decade, many reviews with the phrase “Magnetic nanoparticle” in the title have been published (according to the Web of Science). However, integration of magnetic and the property of fluorescent nanoparticles/QDs in the theranostic applications are not much explored. Herein, we summarize the development of magneto-fluorescent nanoparticles/QDs with regard to synthesis and applications, especially biomedical applications. First, we focus on a series of magneto-fluorescent nanoparticles (MFNPs) synthetic approaches: paramagnetic ions doped QDs, hybrid nanostructure of QDs and MNPs (polymer and silica as carrier), core/shell and heterostructures, QDs with coating of Gd-chelates, magneto-fluorescent heterocrystals and bifunctionalized Janus particles. The fabrication strategies of the above-mentioned MFQDs/MFNPs include self-assembly, phase separation, nucleation growth methods, microfluidic synthesis. After that, the therapeutic applications proposed by bifunctional structures of MFNPs and MFQDs are introduced. We highlight recent developments of biomedical applications based on these particles including photothermal therapy, drug delivery, bioimaging, cancer therapy, and magnetic hyperthermia, and magnetolytic therapy. Lastly, challenges and possible further researches of magnetic nanolights are briefly discussed to attract the attention of not only chemists and materials scientists but also nanomedical therapists to the fast-track of their applications in the biomedical field.

## 2. Magneto-fluorescent nanoparticles (MFNPs)

The strategy to fabricate a material that concurrently holds more than one functional component is an emerging research domain with the potential to have an impact on extensive fields of technology.^43,44^ Those materials are called multifunctional material. Particularly, MFNPs have been acknowledged as a developing group of material that can be employed in advanced applications.^45–47^ MNPs are a class of intelligent materials having magnetic response, large specific surface area, small particle size, and reveal superparamagnetism. On subjection to the external magnetic AC field, MNPs can heat up which opens up visions of hyperthermic features. Also, external magnetic fields could fetch particles to a particular site of interest and thus can play a role in site-specific delivery vector. The combination of magnetic with photoluminescent property in one material unlocks a path towards newfangled nanomaterials.

There are different types of MFNPs: silica coated MFNPs, fluorescently labeled lipid-coated MFNPs, surface engineered fluorescent MFNPs with macromolecular anchors, magneto fluorescent quantum dots, magneto-fluorescent heterocrystals, magnetic and luminescence bifunctionalized Janus particles. The MFNPs could serve as multimodal probe for medical diagnostics, *in vitro* and *in vivo* biolabeling including MRI, fluorescence microscopy and drug delivery systems. Further interesting applications of the MFNPs consist of cytometry, cell tracking, and magnetic separation, which could be simply controlled and monitored using confocal or fluorescent microscopy and MRI.^48,49^ Thus, these intriguing nanostructures have appealed a lot of consideration in recent years.

## 3. Motivation and key challenges for the development of MFNPs

Hybrid nanoparticles which integrate various functionalities including luminescent and magnetic property can provide improved efficacy and versatility with a broad range of potential applications. The combination of fluorescent and magnetic features would be advantageous for MFNPs in different ways such as (i) *in vitro* and *in vivo* biolabeling applications including MRI, fluorescence microscopy (ii) bimodal anticancer therapy: photodynamic and hyperthermic abilities (iii) nanomedicine: therapeutic tool for the treatment of different diseases (iv) nano building blocks for different nanoelectronic and photonic devices employing an external magnetic field to control or arrange the MFNPs and monitor and visualize their position by fluorescence microscope. Although MFNPs are promising for versatile applications, there are also some challenges to overcome in their synthesis procedure. The key challenges are (i) complexity in the fabrication process: multistep preparation and purification, (ii) time consuming (iii) one certain difficulty is the risk of quenching of the fluorophore by magnetic core; also if several fluorescent molecules are attached to the surface of the particles they turn to quench each other (iv) aggregation and instability. The difficulties in fluorescent quenching of the MFNPs can be partially resolved by initial treatment of the fluorophore with spacer or by coating the MNPs with stable shell before the introduction of fluorophore molecule. Another typical problem of MFNPs aggregation can be due to the different kind of interactions between the particles including chemical, magnetic or electrostatic. Herein, in this review, we will discuss the synthesis routes in details which may be employed to offer MFQDs/MFNPs composites and techniques to ensure minimum quenching.

## 4. Magneto fluorescent quantum dots (MFQDs) nanocomposites

Advances in nanoscience and technology revealed new possibilities to incorporate different nanoparticles in one single hybrid nanoarchitecture. Recently, the functional materials based on QDs and MNPs are drawing attention owing to their superiority and potential for the greatest application value.^50–53^ The discussion about the development of different approaches to fabricate MFQDs nanocomposites will be described in details in this review.

### 4.1 Different approaches to integrate magnetic and fluorescence features in QDs

**Fig. 1:**
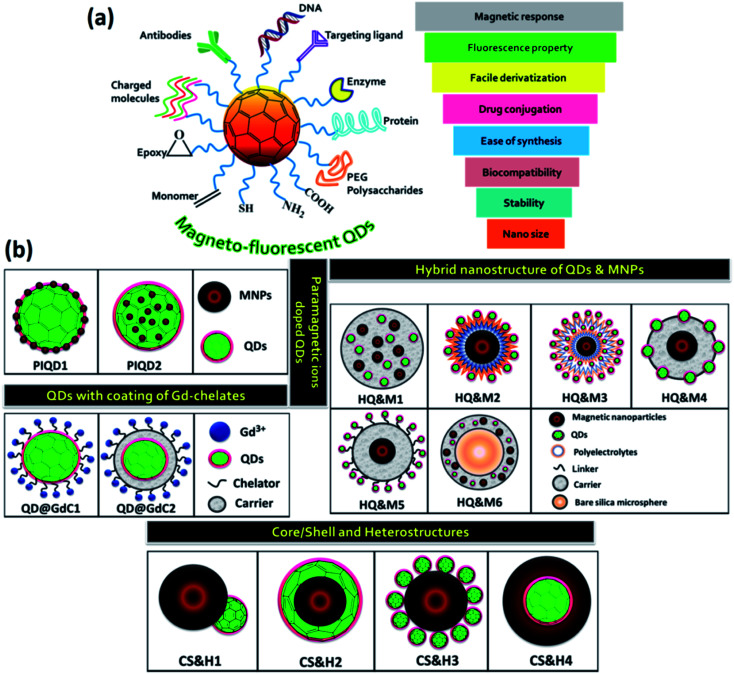
(a) Overview of the unique properties of MFQDs rendering them excellent candidacy for biomedicine, magnetic imaging, and cancer therapeutics. (b) Schematic representation of different types of MFQDs.

Highly fluorescent QDs have emerged as a potential fluorescent label because of their significant optical properties. In comparison to fluorescent dyes, QDs do not have the setback of photobleaching, and their emission colors can be tuned from visible to near-infrared NIR region by varying the size or composition of QDs.^30,54–58^ MNPs are excellent class of materials holding great application values. They behave like superparamagnetic material at room temperature when their size is below a critical level. Such individual MNPs showed large constant magnetic moment. Based on the exciting features of the QDs and MNPs, the composite material revealed promising applications in various field including cancer research, drug delivery, engineering and environmental arena *etc.*Fig. 1a displayed various properties and the surface functionalization of the MFQDs. The synthesis strategies developed for the fabrication of the MFNPs with focus on recent modifications made are discussed in the next section. Pictorial representations of different types of MFQDs are displayed in Fig. 1b.

#### 4.1.1 Paramagnetic ions doped QDs

**Fig. 2:**
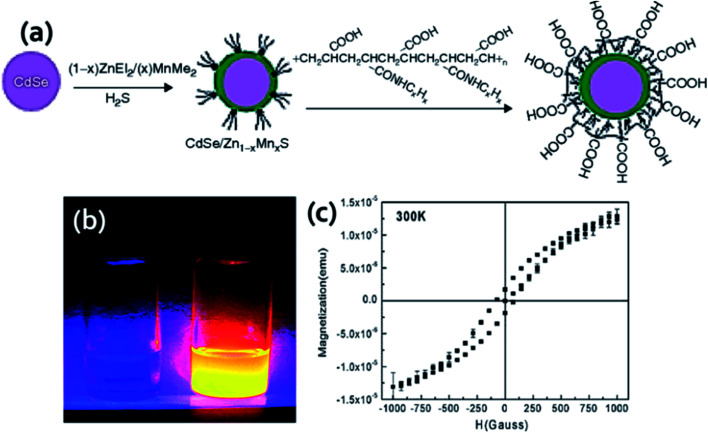
(a) Synthesis procedure for the fabrication of water soluble core/shell CdSe/Zn_1−*x*_Mn_*x*_S. Reproduced from ref. 65 with permission from American Chemical Society, copyright 2007. (b) Fluorescence of yellow-emitting QDs (right) and DI water (left) under 366 nm UV source and (c) magnetization curve for QDs. Reproduced from ref. 70 with permission from American Chemical Society, copyright 2005.

This section discusses the doping approaches of paramagnetic ions into QDs. This method offers a direct mode to unite luminescent and magnetic features into a single nanoparticle. Paramagnetic ions create energy states within the bandgap of QDs, inject energy traps and therefore altered the recombination dynamics and charge separation which decide the emission, lifetime and wavelength of the QDs. Doping with optically active transition metal ions such as Mn^2+^, could modify the photophysical and electronics property of the QDs.^59–61^ In 1994, after Bhargava *et al.* reported the high QY and short fluorescence lifetime of ZnS : Mn^2+^,^62^ extensive research on QDs doped with transition metal ions including paramagnetic ions rapidly augmented. This revealed an entire new research domain for various innovative and interesting applications for paramagnetic ion doped QDs. Incorporation of the dopant ions into the core is a significant aspect. The direct approach involves the growth of QDs in a solution of QDs' precursors and paramagnetic dopant, in the presence of a passivating ligand.^63–69^ The first example we will discuss is of the type of PIQD1 (Fig. 1b) that was reported by Louie *et al.*^65^ Here, the author has developed CdSe QDs (core) which were overgrown with paramagnetic Mn^2+^ ions doped ZnS shell to enhance the paramagnetism on the relaxivity of the surrounding water molecules. The fluorescence QY of the CdSe core was greater by more than 20%. By coating with octylamine-modified poly(acrylic) acid, the core/shell nanoparticles were made water soluble as illustrated in Fig. 2a. The presence of Mn^2+^ ions in the outer layer enabled good interaction with water and thus offered an efficient MRI contrast agent.

The layered QDs with paramagnetic ion doped shell evade the usage of environmentally sensitive organic dyes and get the benefit of stability of QDs emission to degradation or bleaching. This QDs exhibited bright yellow emission under 366 nm UV light and good magnetic properties. Liu and his coworkers for the first time reported the successful incorporation of Mn^2+^ in CuInS_2_ to fabricate MFQDs.^66^ These QDs with no toxic elements showed paramagnetic behavior and tunable fluorescence in a wide window from 542 to 648 nm. As the temperature increases, the size of the nanocrystals core increases from 3.6 to 6.5 nm. EPR study revealed six-line spectrum which confirmed that Mn^2+^ was embedded into the QDs. These MFQDs holds potential applications in photocatalytic field, bioimaging and biomedical arena. Lin *et al.* reported introduction of paramagnetism into QDs without losing QY by capping nanoparticles cores with Mn doped ZnS surface.^67^ The amount of Mn was in the range of 0.2–2.3% and these materials showed high QY in organic solvent. The authors demonstrated magnetic resonance and confocal imaging studies of these nanoparticles and envisioned their use in dual mode optical imaging and as MRI probe. A novel NIR emitting Mn-doped QDs were prepared by Yong and his coworkers.^69^ The CdTeSe/CdS QDs hold outstanding fluorescence and paramagnetic behavior. By surface functionalization of these QDs with lysine, they can be dispersed in aqueous solution, stable in physiological conditions and capable to conjugate with targeting molecules. The structural and elemental analysis endorsed that the magnetism of the QDs was due to the presence of Mn present into the system which acts like a homogeneous dilute magnetic semiconductor. In one report by Santra and his group, 3.1 nm core–shell CdS : Mn/ZnS QDs (PIQD2 type, Fig. 1b) which was fabricated using w/o microemulsion method and coated with silica layer which was additionally modified to obtain primary amine functionalities on the surface for bioconjugation.^70^ Under 366 nm UV light yellow emission was detected (Fig. 2b). Using SQUID the authors conducted magnetic measurement of QDs. The QDs exhibited paramagnetic characteristics with a typical hysteresis curve for a paramagnetic material at room temperature as shown in Fig. 2c. In another approach, Ma *et al.* reported QDs that were grown *via* PECVD method from a GeH_4_/Ar mixed gas under constant flow conditions at 400 °C.^71^ After that, QDs were doped with Mn ions through magnetic sputtering procedure and annealed at 600 °C. These QDs revealed ferromagnetic property at room temperature and 77 K, holding the magnetic behavior as a function of temperature and external magnetic field with average magnetic moment per Mn atom as 2.36 *μ*_B_. ZnS, ZnSe and CdS doped with Mn unveiled emission about 585 nm owing to the Mn d–d transition which is both orbital and spin forbidden resulting in very long lifetime.^60,72^ The long-lived Mn doped QDs are beneficial to boost the efficiency of solar cells. In this context, Santra *et al.* prepared Mn^2+^ doped CdS and successfully improved the QDSC performance.^73^ By boosting the power conversion efficiency to 5%, these QDs revealed competitive potential with other emerging solar cells. Another well-known process for the fabrication of QDs is hot injection technique which could be modified by incorporation of dopant into the reaction mixture.^74,75^ However, using high temperature approach for Mn doped CdSe nanocrystals Bawendi *et al.* found that Mn segregated at the surface of the particles.^76^ This shortcoming of the high temperature reaction was overcome when Norris *et al.* reported the preparation of crystalline QDs ZnSe : Mn nanocrystals that were monodisperse and highly fluorescent.^74^ The confirmation of Mn actually embedded inside the nanocrystal was obtained from optical, EPR, and MCD measurements. This method further indicated that higher Mn doped samples can be obtained using this approach. Furthermore, another approach called cluster method which showed feasible incorporation of Mn^2+^ in CdSe QDs. Here, as precursor organometallic cationic clusters are applied similar to hot injection method but maintained controlled increase to the reaction temperature.^77^ By employing this method, effective doping of both Mn^2+^ and Co^2+^ in CdSe QDs was reported by Gamelin *et al.*^78^

#### 4.1.2 Hybrid nanostructure of QDs and MNPs

In this method, incorporation of magnetic properties in QDs was done by applying carrier material to fabricate hybrid particles in which both fluorescent and magnetic functionalities can be integrated. The key inspiration for the concurrent encapsulation of both QDs and magnetic nanoparticles into carrier capsules was the prospect to generate multifunctional probe which could be addressable by magnetic field and detectable by their fluorescence. Nanoparticles could either be attached to the outside of the carrier material, incorporated into it, or a combination of both. This type of hybrid nanostructure of QDs and MNPs was schematically shown in Fig. 1b. Polymer and silica matrices were applied as carrier material which will be discussed below. In the first section we will focus on hybrid nanostructure using polymer as carrier material and later in the second section we will discuss role of silica as carrier material.

##### Polymer as carrier material

**Fig. 3:**
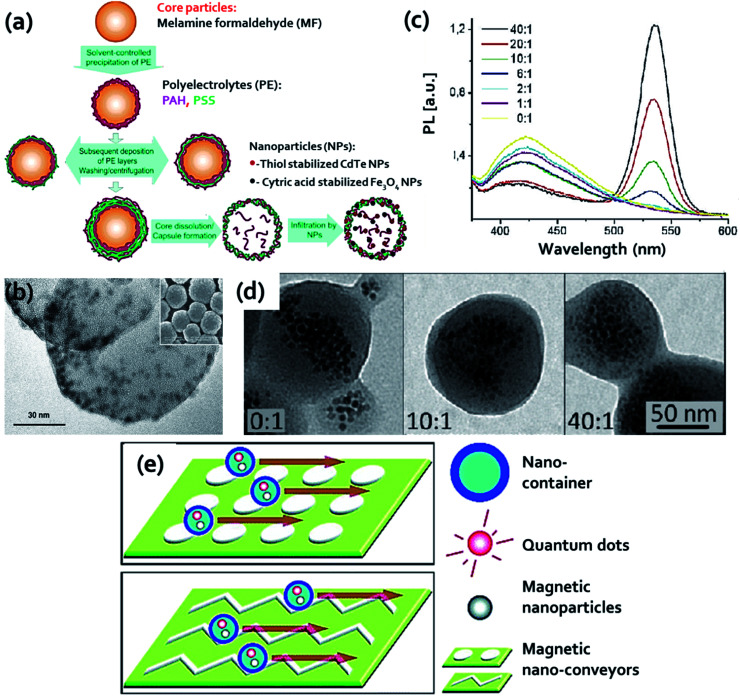
(a) Schematics of fabrication of microcapsules and encapsulation of nanoparticles. Reproduced from ref. 79 with permission from American Chemical Society, copyright 2004. (b) TEM image of both QDs and γ-Fe_2_O_3_ nanoparticles embedded in polymer nanospheres; inset SEM image of hydrazinized styrene/acrylamide copolymer nanospheres. Reproduced from ref. 1 with permission from John Wiley and Sons, copyright 2005. (c) Photoluminescence spectra of the acetonitrile destabilized nanobeads at 350 nm excitation, and (d) TEM images of beads at different QD : MNP ratio, respectively. Reproduced from ref. 80 with permission from American Chemical Society, copyright 2011. (e) Schematic of the nano-conveyor-belt technology. Reproduced from ref. 84 with permission from American Chemical Society, copyright 2010.

In 2004, Rogach and his group first reported polymers as carrier material that have incorporated Fe_3_O_4_ MNPs (8 nm) and CdTe QDs (3–6 nm size, 15–30% QE) into hollow polymer microcapsules (5.6 μm diameter).^79^ The polymer microcapsules contained two oppositely charged PEs, PAA and PAH. The hybrid particle of type HQ&M1 was obtained by the penetration of negatively charged nanoparticles into microcapsules having 10 nm pores. The method used for the synthesis of the microcapsules and the encapsulation process of the nanoparticles was shown in Fig. 3a. The magnetic and fluorescent properties of these hybrid particles were established by aligning the spheres in a magnetic field and probing the imaged under a confocal microscope at 476 nm excitation. Hybrid material that resembles in HQ&M1 (Fig. 1b) was reported by Xie *et al.*, who developed bifunctional nanosphere by co-embedding CdSe/ZnS QDs (3–6 nm) and γ-Fe_2_O_3_ magnetic nanoparticle (5–20 nm) into nanosphere of hydrazinized styrene/acrylamide copolymer.^1^ A modified emulsifier free polymerization was applied to prepare copolymer nanosphere. The hydrophobic moieties of the polymer were located at the interior whereas the hydrophilic functionalities were found to be towards the outer surface of the nanosphere, resulting in the production of hollow hydrophobic cavities. It is clearly obtained from the Fig. 3b that particles were distributed inside the nanospheres with a relatively clean surface. Pellegrino and his coworkers described an approach to fabricate poly(maleic anhydride-*alt*-1-octadecene) embedded CdSe/ZnS QDs and manganese iron oxide nanoparticles of 70–160 nm diameter.^80^ The fluorescent magnetic nanobeads were obtained by adding a destabilizing solvent to the polymer and nanoparticles starting solution. The author also discussed that the selected solvent assists to control the bead size and nanoparticles distribution within the polymer and the fluorescence response of the nanobead could be regulated by changing the relative ratio of QDs and magnetic nanoparticles (Fig. 3c). TEM images of beads at different QDs : MNPs ratio were shown in Fig. 3d. In addition, the surface of the nanobead was decorated with folic acid for specific targeting of cancer cells overexpressing folate receptors. Another interesting method was applied by Yang *et al.*^81^ for encapsulating Fe_2_O_3_ nanoparticles, CdTe QDs, and drug molecule in PCL microcapsules that are resembles in of type HQ&M1 (Fig. 1b). They utilized a facile microfluidic emulsification process where in PCL phase the drug molecule and nanoparticles can be entrapped without fence off into the immiscible phase caused when o/w emulsions are produced by shearing one liquid into a second immiscible one. The size, magnetic, optical and drug release property of the microcapsules were trailored by crosslinking the composite PCL microcapsules with PVA. The next example of a polymer as carrier material that we will be discussed here was reported by Li and his group.^82^ The author demonstrated encapsulation of QDs and MNPs into highly cross-linked carboxylic poly(styrene-*co*-ethylene glycol dimethacrylate-*co*-methacrylic acid) beads (PSEMBs) by an effective technique combining a conventional swelling approach assisted with high-temperature swelling method. Firstly, the MNPs were encapsulated into PSEMBs using conventional swelling process and subsequently QDs were embedded in the MNPs-beads by high temperature swelling approach. QDs embedded into pre-prepared MNPs-PSEMBs afford uniform, stable, and strong fluorescent response and also carry out fast separation. With increasing iron oxide content, noteworthy decrease in the fluorescence response was obtained having the best content in between 5–15 μg mg^−1^. The influence of the order of QDs and MNPs doped into PSEMBs on the magnetic property, fluorescence intensity, and stability of the QDs-MNPs embedded PSEMBs in various pH buffers and organic solvents were assessed. Leaching of the nanoparticles from the polymer beads was studied by suspending QDs and MNPs encapsulated PSEMBs in organic solvents like cyclohexane. Though the hydrophobic QDs have excellent solubility in cyclohexane, insignificant leaching (less than 6%) was detected, indicating superior stability of the beads. The next example of HQ&M1 (Fig. 1b) was developed by Zhou *et al.*^83^ This group proposed a facile and convenient method to produce wheat germ agglutinin-modified trifunctional nanospheres (WGA-TFNS) and surface-expressed with sialic acid and *N*-acetylglucosamine. The existence of wheat germ agglutinin on the surface of WGA-TFNS was established by FTIR analysis, bio-recognition of carboxymethyl chitin-modified QDs, and bacterium *S. aureus*.

The last example of HQ&M1 (Fig. 1b) for polymer as carrier that we will discuss was reported by Winter *et al.*^84^ They described a “nano-conveyer-belt” technology based on MFNPs that allows simultaneous monitoring and movement of nanostructure in a certain direction. This strategy contained two key components: (i) nanocontainers: composed of polymeric micelles (∼35 nm) with hydrophobic core which encapsulate QDs and iron oxide nanoparticles and (ii) nanoconveyors: composed of microfabricated magnetic patterns coupled with electromagnets that offered tunable and high magnetic field gradients required for controlled particle motion. Nanocontainer movement was controlled by nanoconveyers. The encapsulated magnetic nanoparticles permit to propagate the containers which were magnetically manipulated by nanoconveyors as shown in Fig. 3e.

**Fig. 4:**
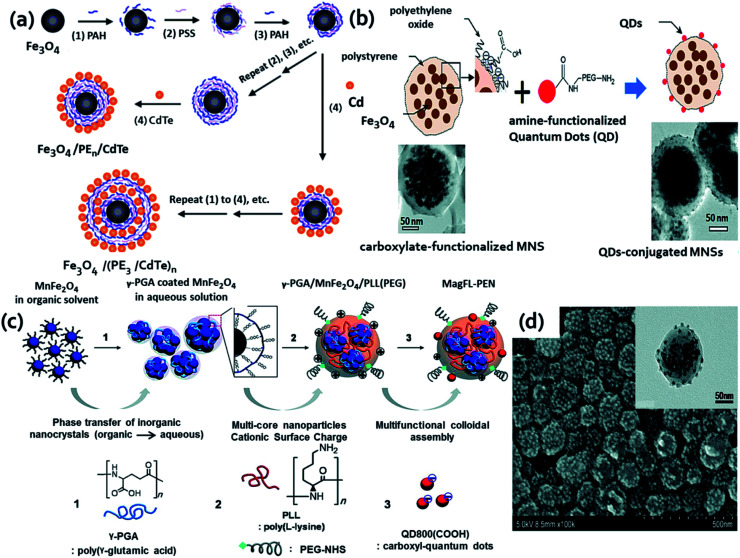
(a) Schematic of layer-by-layer technique to form the magnetic luminescent nanoparticles. Reproduced from ref. 85 with permission from American Chemical Society, copyright 2004. (b) Surface functionalization of magnetic nanosphere (MNSs) conjugation of amine-functionalized QDs to the surface of carboxylate-functionalized MNSs by NHS/EDC coupling method. Below are the respective HRTEM image of the MNSs before (left) and after (right) the surface modification. Reproduced from ref. 87 with permission from American Chemical Society, copyright 2010. (c) Schematic presentation of the fabrication of magnetofluorescent polyelectrolyte nanocomposites (MagFL-PEN) through electrostatic assembly and (d) SEM and TEM (inset) images of MagFL-PEN after adsorption of QD800(COOH). Reproduced from ref. 88 with permission from American Chemical Society, copyright 2011.

In a report by Bai and coworkers, they explained the LBL strategy to fabricate water soluble MFNPs (Fig. 1b HQ&M2 and HQ&M3) using polymer as a carrier as shown in Fig. 4a.^85^ By this method, the Fe_3_O_4_ nanoparticles (8.5 nm) were encapsulated by alternating layers of negatively and positively charged PEs. For the controlled deposition of CdTe QDs/PEs multilayers, Fe_3_O_4_ nanoparticles were employed as a template. By varying the deposition cycles of PE interlayers and CdTe QDs/PE multilayers, Fe_3_O_4_/PE_*n*_/CdTe and Fe_3_O_4_/(PE_3_/CdTe)_*n*_ were synthesized, respectively. The benefit of this method was that the distance between CdTe QDs and Fe_3_O_4_ could be tuned by introducing different PEs layers. It was noticed that the fluorescence intensity of the QDs was increased with increasing interparticle distance. In addition to the PL property, these nanoparticles unveiled excellent magnetic properties and were simply separated from the solution employing a permanent magnet. The authors elucidated this study as a combination of two effects: first one was the distance-dependent quenching of the CdTe QDs fluorescence by the Fe_3_O_4_ particles, and the second effect was caused at larger distances when the surface area was increased resulting in increasing attachment of CdTe QDs amount.

The next example that is like HQ&M2 (Fig. 1b) was illustrated as fast and simple fabrication of Fe_3_O_4_-PEI-QDs by Yu and his groups.^86^ The synthesis process involved assembling hydrophobic TOPO capped CdSe@ZnS QDs (4 nm) on PEI coated Fe_3_O_4_ nanospheres (50 nm). PEI was used for the realization of multifunctionality, as well as attaching TOPO capped CdSe@ZnS QDs onto Fe_3_O_4_ nanoparticles and therefore modifying the surface properties of QDs from hydrophobic to hydrophilic along with avoiding the formation of agglomeration. These MFNPs displayed good colloidal stability, fluorescent and magnetic properties. The next example of polymer as carrier for both QDs and MNPs of HQ&M4 (Fig. 1b) that we will discuss here was developed by Shi and his groups.^87^ In this study, the authors described multifunctional nanosystem with superparamagnetic Fe_3_O_4_ nanoparticles (∼10 nm) embedded inside a spherical polystyrene matrix (∼150 nm) and QDs with emissions ∼800 nm (NIR range) conjugated onto the surface of the nanocomposite (Fig. 4b).

By the conventional EDC/NHS coupling technique, the amine functionalized QDs were covalently conjugated to the surface of carboxylate-functionalized, polyethylene oxide modified MNPs. As evident from the Fig. 4b, the surface modification resulted in ruffled and dark spotted surface after QDs conjugation. Further the drug storage was executed by loading paclitaxel onto the surface of the composite nanostructure by applying poly(lactic-*co*-glycolic acid) layer. Also, the authors previously reported similar coupling approach to conjugate QDs to polystyrene–Fe_3_O_4_ composite for *in vivo* imaging applications.^89^ An improved LbL assembly was employed to yield fluorescent magnetic dual encoded nanospheres which exhibited good monodispersibility, enormous encoding capacity and well-preserved features of both magnetic nanoparticles and QDs.^90^

Here, the authors demonstrated that QDs and nano-γ-Fe_2_O_3_ can be controllably assembled on the surface of poly(styrene/acrylamide) copolymer nanospheres without capacity limitation. Without any pretreatment, TOPO-capped CdSe/ZnS QDs and oleic acid coated magnetic γ-Fe_2_O_3_ particles were directly and controllably assembled on branched PEI coated nanospheres. These dual-encoded nanospheres with various fluorescence emissions and magnetic susceptibility were created due to the tunable coating of QDs and γ-Fe_2_O_3_ nanoparticles along with control fluorescent emissions of deposited QDs. The next example of MFNPs that look like HQ&M4 (Fig. 1b) was illustrated by Kim and coworkers.^88^ They developed a chemical strategy to obtain MR/NIR dual modality probe based on polyelectrolytes, metal doped multiple core MNPs, and fluorescent QDs. The synthesis of these MFNPs was reproducible since most of the steps were conducted by means of ionic interaction between functional inorganic nanoparticles and polyelectrolyte under mild aqueous conditions. The entire synthesis procedure was schematically shown in Fig. 4c. As clearly evident from the SEM (Fig. 4d) and TEM (inset of Fig. 4d) images that satellite of MnFe_2_O_4_ nanoparticles were encapsulated inside MagFL-PEN and the QDs were coated on to the surface. Magnetic fluorescent molecularly imprinted polymers (MIPs) were prepared using Fe_3_O_4_–C-dots by one step method as fluorescent source and supporting matrix, dopamine which can undergo self-polymerization in alkaline condition to form thin polydopamine film on various types of materials, bovine hemoglobin (BHb) as the template molecule. The magnetic C-dots@MIPs served as specific binding sites for BHb and good fluorescence and magnetism properties.^91^ In brief, researchers are putting great efforts for the preparation of MFNPs using polymers as carrier which were reported elsewhere.^92–96^

##### Silica as carrier material

**Fig. 5:**
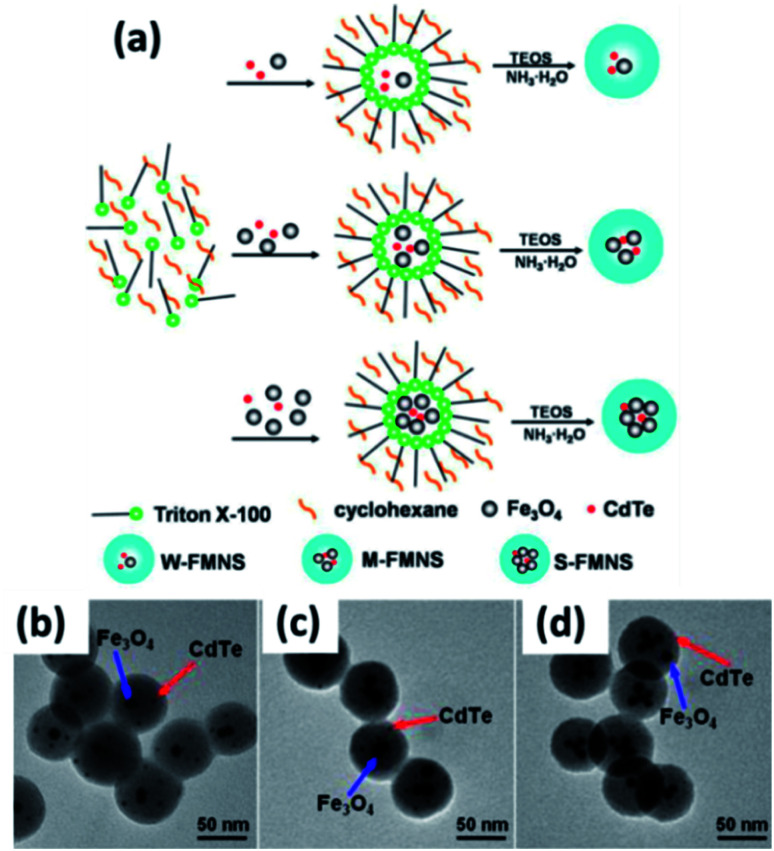
(a) Schematic of the preparation of (CdTe/Fe_3_O_4_)@SiO_2_ FMNS with various magnetic potentials *via* reverse microemulsion method. TEM images of (CdTe/Fe_3_O_4_)@SiO_2_ FMNS: (b) W-FMNS, (c) M-FMNS, and (d) S-FMNS. Reproduced from ref. 99 with permission from American Chemical Society, copyright 2014.

In addition to the polymer, silica has also served as a carrier material for QDs and MNPs that resembles HQ&M1 (Fig. 1b) and was described by Yi *et al.*^97^ The authors stated a novel hybrid material comprising QDs and MNPs encapsulated in a silica shell. γ-Fe_2_O_3_ and CdSe QDs were used as magnetic and fluorescence entity, respectively. γ-Fe_2_O_3_ and CdSe QDs were introduced to Igepal CO-520 dispersed in cyclohexane. The mixture was vortexed and NH_4_OH was supplemented to form reverse microemulsion. Further, TEOS was added and the reaction was continued for 48 h, resulting in the formation of SiO_2_/MNPs–QDs nanocomposite. Kim *et al.* reported monodispersed magnetite nanocrystals and QDs encapsulated in uniform pore sized mesoporous silica spheres (average particle size ∼150 nm).^98^ These nanocomposite were applied for uptake, and controll release of ibuprofen while the rate of release was governed by the surface property of the silica spheres. The next example of HQ&M1 (Fig. 1b) of silica as carrier that we will discuss was reported by Song and his coworkers.^99^ They fabricated magnetic encoded fluorescent CdTe/Fe_3_O_4_@SiO_2_ nanospheres through a reverse microemulsion technique presented in Fig. 5.

The simultaneous encapsulation of QDs and Fe_3_O_4_ nanoparticles into silica shell was carried out to obtain MFNPs by means of hydrolysis and condensation of TEOS under ammonia catalysis. The degree of magnetism of the MFNPs were controlled by changing the initially concentration of Fe_3_O_4_. These MFNPs showed small size (about 60 ± 5 nm) and high magnetic capture efficiency, greater than 90% for every type of MFNPs with various magnetic susceptibilities (W-weak, M-moderate, and S-strong) by applying fluorescently labeled IgG-FMNS conjugates. The next example of mesoporous Fe_3_O_4_/SiO_2_/CdTe MFNPs resembles to HQ&M5 (Fig. 1b) was reported by Yin *et al.*^100^ The synthesis procedure of mesoporous Fe_3_O_4_/SiO_2_/CdTe MFNPs involved two steps. In the first step, TEOS as silica source was used for the controlled growth of mesoporous silica coating on the oleic-acid-stabilized Fe_3_O_4_ surface. This step involved CTAB as cationic surfactant template and TMB as pore swelling agents. The next step involved decoration of luminescent CdTe QDs on the mesoporous Fe_3_O_4_/SiO_2_ using APS as a linker. These nanoprobe unveiled superparamagnetism at room temperature, strong photoluminescent property, and potential drug delivery vector. Therefore, these MFNPs can simultaneously offer three purposes as fluorescence tracking, magnetic separation, and drug delivery. TEM images of mesoporous Fe_3_O_4_/SiO_2_ and mesoporous Fe_3_O_4_/SiO_2_/CdTe nanocomposite showed they are uniform and separated from one to another.

**Fig. 6:**
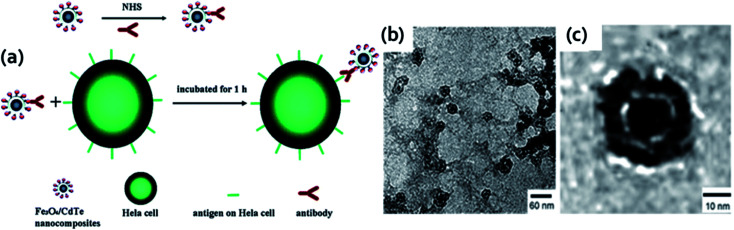
(a) Schematic design of immuno-labeling by Fe_3_O_4_/CdTe nanocomposites. (b and c) TEM image of Fe_3_O_4_/CdTe MFNPs. Reproduced from ref. 2 with permission from American Chemical Society, copyright 2010.

A new strategy for the synthesis of HQ&M5 (Fig. 1b) MFNPs (diameter ∼30 nm) was proposed by Xu *et al.*^2^ where TGA stabilized QDs were covalently linked to and assembled around thiol functionalized silica-coated superparamagnetic Fe_3_O_4_ core–shell nanoparticles. Firstly, sol–gel method was applied to coat the Fe_3_O_4_ nanoparticles with silica shells and followed by functionalized with thiol groups. After that, multiple TGA stabilized CdTe QDs were chemically conjugated to the silica coated Fe_3_O_4_ surface by the reaction between thiols functionalities on QDs and silica. The carboxyl groups present on the surface of the MFNPs were chemically active and permits them for further bioconjugation with biomolecules for instance BSA and anti-CEACAM8. Therefore, the Fe_3_O_4_/CdTe hybrid nanostructures were successfully utilized as fluorescent marker for imaging of HeLa cells (Fig. 6a). The TEM images (Fig. 6b and c) of the Fe_3_O_4_/CdTe MFNPs confirmed that multiple small sized QDs were coated around the surface of the individual silica-coated Fe_3_O_4_ core–shell nanoparticle. These MFNPs displayed good fluorescent and magnetic features for potential applications in fluorescent tracking as well as magnetic separation. Another kind of MFNPs of HQ&M6 (Fig. 1b) was proposed by Insin *et al.*^101^ These hybrid particles were synthesized by incorporation of CdSe/CdZnS QDs and γ-Fe_2_O_3_ into a silica shell around prefabricated silica microspheres. The hybrid particles of ∼500 nm showed uniform integration of QDs and MNPs into the shell with narrow size distribution.

The aforementioned examples indicate that hybrid particles using polymers or silica as carrier materials can be fabricated in many flavors. This approach involved several clear benefits including the control over the distance between the QDs and MNPs, high payload of QDs and MNPs that could be embedded, tunability between the ratio of both the nanoparticles. The surface chemistry of polymer and silica shells was well-developed, enabling further bio-functionalization of these carriers. Additionally, other examples of fabrication of MFNPs using silica as carrier were reported elsewhere.^102^

#### 4.1.3 Core/shell and heterostructures of magnetic and QDs materials

In this section, we will discuss about the nanocrystals that bear a resemblance to the structure represented as in Fig. 1b. Here, the QDs and the MNPs were fused together to produce either a core/shell or heterostructure. In spite of large lattice mismatch between semiconductor nanocrystals and MNPs, enormous efforts had been taken up by the researchers to unite the both within one nanocrystal, though the attachment mechanism has not been yet completely resolved.

**Fig. 7:**
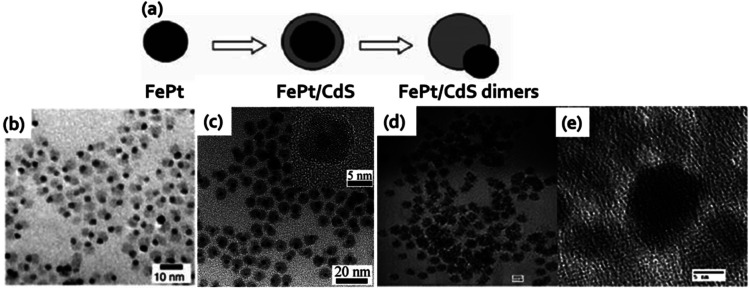
(a) Schematic approach used for the preparation of FePt–CdS heterodimers. (b) TEM images of FePt–CdS heterodimers. In the dimers the light gray dots were CdS, whereas the darker ones were FePt nanoparticles. Reproduced from ref. 103 with permission from American Chemical Society, copyright 2004. (c) TEM image of FePt@CdSe core–shell nanocrystals (HRTEM image in inset). Reproduced from ref. 105 with permission from American Chemical Society, copyright 2007. (d) TEM image of QD-magnetic beads core–shell nanoparticles. (e) HRTEM image of a single magnetic bead coated with QDs. Reproduced from ref. 106 with permission from American Chemical Society, copyright 2004.

The first example that will be discussed here is of CS&H1 was reported by Gu *et al.*^103^ This group demonstrated a one-pot preparation strategy to produce heterodimers of nanoparticles by considering the benefit of lattice mismatching and selective annealing at a relatively low temperature. Briefly, amorphous CdS was deposited on the FePt nanoparticles surface to yield a metastable core–shell structure wherein upon heating the CdS was transformed from amorphous to crystalline phase. Due to the lattice mismatch between CdS and FePt nanoparticles and the surface tension when they were dispersed in solvent, core–shell nanoparticles of FePt@CdS transformed into heterodimers of FePt and CdS nanoparticles (Fig. 7a). TEM images of FePt–CdS heterodimers was shown in Fig. 7b. The final particles size was less than 10 nm and revealed both fluorescence and superparamagnetism with a blocking temperature of 11 K. The fluorescence emission of the heterodimers was at 438 nm which was consistent with similar sized CdS QDs and showed QY of 3%. Selvan *et al.* illustrated a facile strategy to prepare heterodimers or a homogeneous dispersion of QDs around the magnetic cores.^104^ The CdSe QDs were grown onto prefabricated cores of Fe_2_O_3_ magnetic nanoparticles at high temperature around 300 °C in the presence of an organic surfactants resulting in the production of heterodimers or a homogeneous dispersion of CDSe QDs around the Fe_2_O_3_ magnetic cores. Later Shim *et al.* demonstrated a careful study about different factors contributing to the structural diversification of Fe_2_O_3_/CdS system. Their study explained how the maximum number of heterojunctions that can generate was dependent on the seed nanocrystals size and about how the resulting nanocrystals heterostructure morphology was influenced by the growth rate. Xu *et al.* reported a study where this group has developed core–shell nanostructures (CS&H2) containing FePt core (∼3 nm) and CdSe shell (3–5 nm).^105^ To a reaction mixture of FePt nanoparticles, addition of Cd(acac)_2_ resulted in the generation of FePt@CdO core–shell intermediates followed by incorporation of chalcogens afforded FePt@CdX (X denoted Se or S) core–shell nanostructure. TEM images (Fig. 7c) evidently showed the monodispersed FePt@CdSe core–shell nanostructure. At room temperature, the QY of FePt@CdSe core–shell nanostructure was 7.5–9.7% while for FePt@CdS the value was little lower at 2.3–3.5%. The comparatively low QYs were described due to the partial quenching of FePt cores. The superparamagnetism characteristics of FePt core was well preserved derived from the low blocking temperature of 13 K and 14 K for FePt@CdS and FePt@CdSe core–shell nanostructure, respectively. Rosenzweig *et al.* described the fabrication of novel nanoparticles comprise of superparamagnetic core γ-Fe_2_O_3_ and CdSe/ZnS QDs shell.^106^ Thiol chemistry was applied to bind the QDs to the surface of magnetic beads that looks like CS&H3. By the formation of thiol-metal bonds, single layer of QDs was bound to the surface of magnetic beads (thiol modified) to yield MFNPs. The TEM and HRTEM images of the nanoparticles were shown in Fig. 7d and e, respectively. The average diameter of the particles was 20 nm along with about 15% size distribution. These nanoparticles have potential applications in bioanalytical assays including luminescence detection and magnetic separation.

**Fig. 8:**
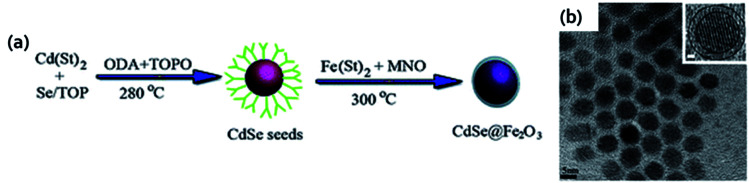
(a) Scheme for the synthesis of CdSe@Fe_2_O_3_ core–shell nanoparticles and (b) HRTEM image of the MFNPs. Reproduced from ref. 107 with permission from Royal Society of Chemistry, copyright 2011.

The next example that we will discuss that resembles CS&H4 proposed by Zhang and group who reported one-step seeded-growth strategy for the synthesis of CdSe@Fe_2_O_3_ core/shell nanoparticles.^107^ Firstly, they prepared (Cd(St)_2_) and then CdSe QDs were treated with TOPO. The TOPO coated CdSe QDs then played as nucleation site for the generation of Fe_2_O_3_ shell around the QDs core upon (Fe(St)_2_) oxidation. The schematic description of the entire synthesis procedure was shown in Fig. 8a. The size of CdSe@Fe_2_O_3_ core/shell MFNPs was increased to ∼9 nm than CdSe nanoparticles (∼7 nm) which indicated the Fe_2_O_3_ coating around the CdSe (Fig. 8b). Analysis of fringes of these MFNPs unveiled the QDs core was coated by ∼1 nm layer of Fe_2_O_3_ which was shown in the inset of the HRTEM image. The next example of core/shell MFNPs that we will discuss here was synthesized by Trinh *et al.*^108^ The authors fabricated FePt@CdSe core–shell nanoparticles *via* addition of precursor materials sequentially and used tetraethylene glycol as reducing agent and solvent. The core–shell nanoparticles were obtained over an extensive temperature range between 240–300 °C. The composition and size of the core were altered by varying the surfactant to metal precursor's ratio and the feeding ratio of the precursors, respectively. Through rational control of the total amount of Cd and Se, the CdSe shell thickness could be tuned between 1–8.5 nm. These MFNPs with 4.3 nm core size and 2.5 nm shell thickness revealed superparamagnetic behavior at room temperature with blocking temperature at 55 K and fluorescence emission was detected at about 600 nm.

A very interesting concept of co-assembling MNPs with QDs to acquire colloidal core–shell MFNPs was proposed by Bawendi and groups.^51^ The authors termed them as supernanoparticles (SPs) which contained superstructure of closely packed magnetic core and surrounded by shell of QDs. To achieve biocompatibility, high colloidal stability and multipurpose surface functionality, a thin layer of silica was coated around these core–shell superstructures. After surface PEGylation, these SPs were manipulated magnetically inside living cells and simultaneous optical monitoring. Hydrophobic CdSe–CdS core–shell QDs of size 9.0 ± 0.4 nm and superparamagnetic Fe_3_O_4_ MNPs of 5.9 ± 0.3 nm size were mixed and transferred to aqueous solution by applying surfactant DTAB followed by rapid injection of the resultant micelle solution into PVP ethylene glycol solution. PVP stabilized SPs were obtained by centrifugation after 30 min of stirring. The TEM analysis revealed that the SPs had ∼120 nm average diameter and comprised of both MNPs and QDs. Scanning TEM elemental mapping also confirmed the core–shell structure of the nanoparticles.

Later, the core–shell MFNPs having biogenic magnetite as core and C-dots as shell were developed by Markova *et al.*^109^ Similar kind of core–shell nanocomposite of C-dots and MFe_2_O_4_ (where M referred to Mn, Zn and Cu) was also reported by Guo *et al.*^110^ Recently, Fe_3_O_4_–CdSe core–shell MFNPs (CS&H2) with core (diameter 10 nm) and shell (thickness 2 nm) was developed by Liu and coworkers through controlled sequential reactions.^111^ These core–shell MFNPs revealed superparamagnetic behavior with high susceptibility at room temperature and two emission center fluorescence behavior. Also, Mozafari and group reported dual core–shell nanostructure Fe_3_O_4_–Cds–Zn nanocomposite where the authors firstly prepared Fe_3_O_4_ as magnetic core using hydrothermal method and followed by preparation of cadmium sulfide doped with Zn as dual shell.^112^ Then, metal doped Fe_3_O_4_–Cds nanocomposites were fabricated through a fast chemical method. The other instances of the core/shell and heterostructures of magnetic and QDs materials were reported somewhere else.^113,114^

#### 4.1.5 QDs with coating of Gd-chelates

The next category that will be discussed here is QDs coated of chelates (organic complexes) of Gd. The lanthanide ion Gd^3+^ has high magnetic moment, symmetric electronic ground state and therefore they are extensively used for application like bioimaging and MRI contrast agent. Though it is well known that Gd^3+^ is toxic and therefore to decrease the toxicity and increase stability surface-chelated Gd^3+^ ions are complexed in organic chelates that coordinate to the paramagnetic ions through an ionic interaction. Either covalently or noncovalently dye molecules have been anchored to the complexes to introduce luminescent features to the paramagnetic chelates. Another strategy to attain bimodality is by attaching paramagnetic chelates to QDs both covalently and non-covalently of which some studies will be discussed herein. In this section, we will discuss about the nanocrystals that bear a resemblance to the structure represented as in Fig. 1b.

**Fig. 9:**
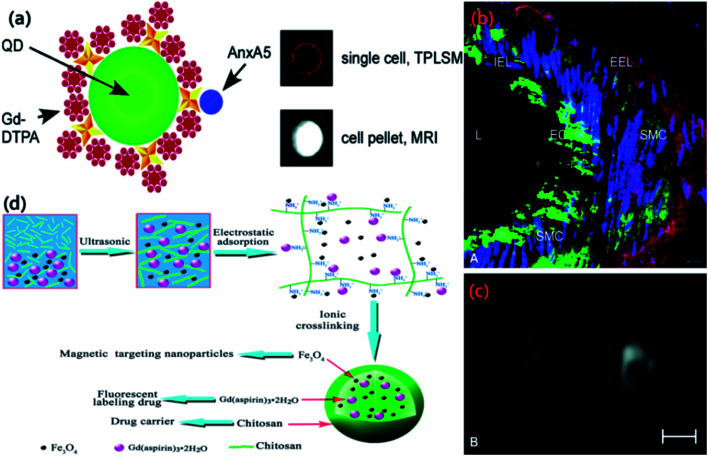
(a) Nanoparticle with the biotinylated Gd-wedge, containing eight Gd-DTPA complexes each (AnxA5-QD-Gd-wedge). Green: QD; yellow: streptavidin; red dot: Gd-DTPA; red star: lysine-wedge; blue: AnxA5. (b) TPLSM image of a damaged murine carotid artery (*ex vivo*), displaying high uptake of green-emitting, annexin A5-conjugated QDs with a Gd-wedge coating in ECs and SMCs. EC: endothelial cells; SMC: smooth muscle cells; L: lumen; EEL: external elastic lamina; IEL: internal elastic lamina. Red: eosin, labeling elastin laminae; blue: syto41, labeling cell nuclei. (c) The same damaged artery revealed brighter contrast in a transversal MR image (right) compared to an undamaged control artery (left). Reproduced from ref. 118 with permission from American Chemical Society, copyright 2007. (d) Schematic diagram of preparation of chitosan microsphere incorporated with Gd(aspirin)_3_·2H_2_O and Fe_3_O_4_. Reproduced from ref. 119 with permission from Elsevier, copyright 2014.

**Scheme 1:**
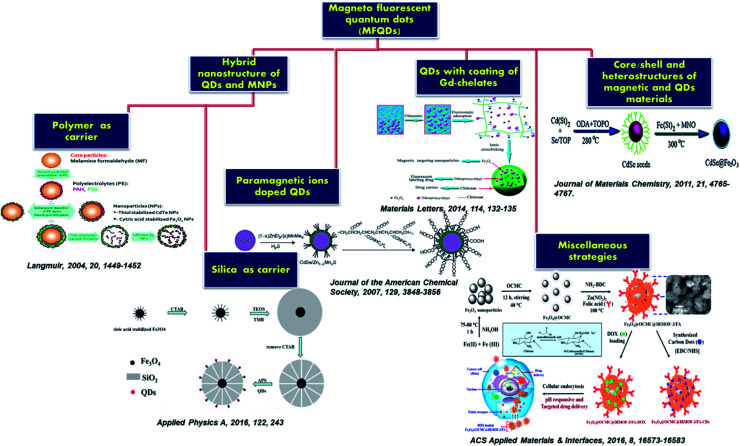
Different types of MFQDs and their synthesis procedure.

The first example of QDs with coating of Gd-chelates that we will be discussed here was demonstrated by Mulder *et al.*, who applied lipidic micelle noncovalently surrounding of the QDs to incorporate Gd-DTPA complexes.^115^ Earlier it was reported that encapsulating QDs in the hydrophobic interior of lipidic micelles their bioapplicability could be rendered. The authors adapted this method to coat QDs with pegylated (PEG-DSPE) and paramagnetic (Gd-DTPA-BSA) lipids. The QDs of type QD@GdC1 showed bright and narrow emission at around 560 nm and ionic relaxivity, *r*_1_, of nearly 2000 mM^−1^ s^−1^ per QDs. The *r*_1_ of Gd-DTPA-BSA was 12.4 mM^−1^ s^−1^, which was higher than that of free Gd-DTPA due to the lower tumbling rate of the Gd-DTPA-BSA complex in comparison to free Gd-DTPA. The particles were appropriate for *T*_1_-weighted imaging since the *r*_2_/*r*_1_ ratio was 1.5 (*r*_2_ was 18 mM^−1^ s^−1^). The QDs were covalently functionalized with αvβ3-specific RGD peptides, which is overexpressed on both the surface of angiogenic endothelial cells and tumor cells. Later this author also reported highly monodisperse silica particles with QDs incorporation in the center and paramagnetic lipid coating of Gd-DTPA-DSA to facilitate the bimodality features and enabled target specific character by conjugating multiple αvβ3-integrin-specific RGD-peptides for endothelial cells.^116^ In another study, QDs encapsulated in paramagnetic micelle for both MRI and optical imaging was used. Further the QDs were conjugated with annexin A_5_ protein molecules for targeting apoptotic cells.^117^ A similar architecture that resembles to type QD@GdC2 was proposed by Chen and group by applying ultrathin silica shell around CdSe/ZnS QDs.^55^ The 2–3 nm thin silica shell was covalently linked to Gd^3+^ ions chelator, tetraazacyclododecanetetraacetic acid (DOTA). The resulting complex having diameter of 8 to 15 nm was soluble in high ionic strength buffers in the pH range 4–11. The author described that the benefits of this technique were the simplicity of the synthetic route, the flexibility in composition, and the high ionic relaxivity *r*_1_ of 23 mM^−1^ s^−1^. Next example (type QD@GdC1) that we will discussed was proposed by Reutelingsperger and coworkers.^118^ The authors presented intensely fluorescent QDs based nanoparticles (Fig. 9a) for cell death visualization and activated platelets with MRI and fluorescence imaging. Due to increased gadolinium-DTPA loading, the nanoparticles unveiled large MR relaxivity (*r*_1_) of 3000–4500 mM^−1^ s^−1^ per nanoparticle for both anatomic and subcellular imaging. Moreover, targeted labeling was implemented through mechanically injured on murine carotid artery *in vivo* by endothelial denudation with a metal wire. This injury resulted in an overexpression of PS (phosphatidylserine, binds to annexin A5). The damaged artery revealed high uptake of annexin-5-conjugated QD-Gd-wedge particles (green-emitting) visualized (Fig. 9b) by two-photon laser scanning microscopy (TPLSM). The undamaged control artery scarcely displayed any labeling which confirmed the target specificity. In comparison to the control artery, bright signal for the damaged artery was observed (Fig. 9c), endorsing the efficacy of the bimodal nanoparticles. In a report Fan and group prepared fluorescent-labeling drug Gd(aspirin)_3_·2H_2_O by covalently binding Gd^3+^ and aspirin. Gd(aspirin)_3_·2H_2_O and Fe_3_O_4_ were further incorporated into chitosan microsphere to enable bimodality of chitosan microsphere and act as drug delivery vehicle (Fig. 9d).^119^ In another approach, Jing and his group developed Gd(iii) chelate functionalized carbon quantum dots [Gd(iii)/CQDs] for multimodal imaging agent by pyrolysis of gadopentetate monomeglumine.^120^ Here, the precursor gadopentetate monomeglumine served the carbon source as well as Gd(iii) source in the formation of CQDs. Gd(iii)/CQDs with QY of 8.9% was nontoxic to HeLa cells and showed superparamagnetic nature at room temperature having the *r*_1_ ∼ 6.4 mM L^−1^ S^−1^. Similar type of technique (reaction temperature 250 °C) was also reported to fabricate Gd-containing CQDs by mixing gadopentetic acid into Tris base and betaine hydrochloride followed by pyrolysis.^121^ Other approaches were also attained like microwave-assisted polyol method,^122^ hydrothermal method^123,124^ to achieve Gd containing QDs which served as both fluorescence and MRI purposes. Different types of MFQDs nanomaterials and their synthesis process was schematically depicted in Scheme 1.

#### 4.1.6 Miscellaneous strategies

**Fig. 10:**
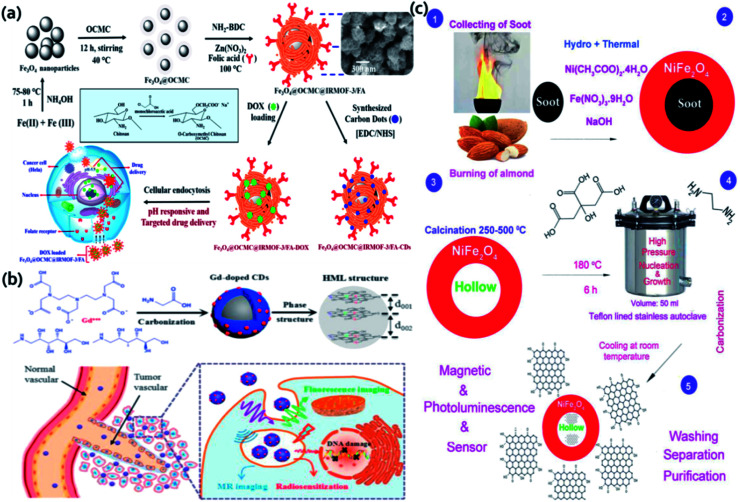
(a) Schematic representation of the synthetic method for the magnetic NMOFs. Reproduced from ref. 126 with permission from American Chemical Society, copyright 2016. (b) Synthesis approach of Gd-doped C-dots by hydrothermal treatment of GDPA and glycine and following applications. Reproduced from ref. 127 with permission from Elsevier, copyright 2017. (c) Preparation of C-dot and hollow magnetic nanocomposite. Reproduced from ref. 129 with permission from Elsevier, copyright 2019.

In this section, we will discuss about some miscellaneous synthesis approaches for the synthesis of QDs containing magnetic nanoparticles apart from the above classifications. The first example that we will discuss was reported by Bhaisare *et al.*^50^ In this work the author prepared amine functionalized magnetic C-dots. Fe_3_O_4_ MNPs were synthesized and mixed with 4% acetic acid chitosan solution and kept in stainless steel autoclave at 180 °C, 12 h for the reaction. After the reaction, the product was purified and collected by external magnet and dried under vacuum for 12 h at 55 °C. This hybrid material was highly sensitive for the fluorescent detection of bacteria. The limit of detection for *E. coli* and *S. aureus* was found to be 3.5 × 10^2^ and 3 × 10^2^ cfu mL^−1^, respectively. Magnetic CoFe_2_O_4_–C-dots nanocomposite was reported by Shahla *et al.*^125^ The C-dots was prepared from inexpensive turmeric precursors. The magnetic nanocomposites were prepared by hydrothermal treatment of cobalt ferrite and turmeric solution for 24 h at 200 °C. The influences of temperature and time on the morphology and particles size of the nanocomposites were studied. The magnetic nanocomposite revealed fluorescence under UV light and showed ferromagnetic property by vibrating sample magnetometer. Next Wang developed hybrid MNPs comprising Fe_3_O_4_ nanoparticles in the core and C-dots in the porous carbon shell.^52^ The author ensured that enough oxidizing agent H_2_O_2_ present in the reaction medium so that C-dots can be produced *in situ* in the porous shell from the oxidation and decomposition of the ferrocene. This hybrid MNPs unveiled good photostability, NIR photothermal effect, aqueous dispersibility, high drug loading and MRI properties. In another report Ding and his group fabricated Cd free Mn doped QDs with Zn gradient CuInS_2_ core and ZnS outer shell.^53^ The synthesis method involved three steps: synthesis of fluorescent CuInS_2_ seeds, particle surface coating of ZnS, and the Mn-doping of the ZnS shells. ZnS shell increased the fluorescence of the core as well as prevents the core from fluorescence quenching due to Mn doping. The hydrophobic CuInS_2_@ZnS : Mn QDs were transferred into water through ligand exchange reaction by replacing 1-dodecanethiol ligand with DHLA-PEG ligand. Additionally, *in vivo* fluorescence and MR imaging demonstrated that the PEGylated CuInS_2_@ZnS : Mn QDs could target both subcutaneous and intraperitoneal tumors *in vivo*. Chowdhuri *et al.* hybrid magnetic nanoscale metal organic frameworks (NMOF) which revealed good stability and biocompatibility (Fig. 10a).^126^ At first, super paramagnetic Fe_3_O_4_ particles were coated with *O*-carboxymethyl chitosan (OCMC) which can be used for pH-responsive drug release at the acidic tumor sites. Then IRMOF-3 was developed with encapsulation of folic acid in one-pot at the Fe_3_O_4_@OCMC nanoparticle surface. Last, DOX was conjugated into NMOFs by physical encapsulation method.

These folate targeted magnetic NMOF unveiled specific cell internalization toward HeLa cells (folate-overexpressed) in comparison to normal L929 cells. In a report Gd-doped C-dots (Fig. 10b) were developed by hydrothermal treatment of Gd-DTPA and glycine.^127^ Gd-DTPA was used as Gd source and glycine as the passivating agent. Gd-doped C-dots could reduce Gd leakage even under complex biological environments by virtue of the inertness of carbon cages. These Gd-doped C-dots have high potential as MRI contrast agents *in vivo* and radiotherapy, good biocompatibility and enhanced longitudinal relaxivity rate (*r*_1_) of 6.45 mM^−1^ S^−1^. Similar hydrothermal method was also stated to fabricate nitrogen and cobalt doped C-dots (N,Co-C-dots) using citric acid, CoCl_2_·6H_2_O, and diethylenetriamine and used for the detection of cholesterol and uric acid in human blood serum.^128^ Another interesting report based on magnetic hollow NiFe_2_O_4_–C-dots nanocomposite (Fig. 10c) was illustrated by Shahla *et al.*^129^ Carbon nano-templates were synthesized from soot of burning almond followed by synthesis of NiFe_2_O_4_ nanoparticles on the carbon templates. To prepare hollow structures, the product was calcinated 500 °C for 2 h to remove the carbon templates. C-dots were fabricated on the hollow nickel ferrite cores by hydrothermal treatment of ethylene diamine and citric acid at 180 °C for 6 h. These nanocomposites were applied for the detection of *Pseudomonas aeruginosa* bacteria.

1-Ethyl-3-(3-dimethylaminopropyl)-carbodiimide/*N*-hydroxysuccinimide (EDC/NHS) coupled reactions are highly efficient and widely used for biomolecule conjugation. Using this technique Pramanik *et al.* developed blue/red fluorescent magneto-C-dots nanoparticles.^130^ Firstly, they prepared carboxylic acid functionalized MNPs by coprecipitation technique using ferric chloride and 1,6-hexanedioic acid. After that EDC mediated esterification was applied to yield MNPs attached with fluorescent C-dots. These amide-coupled fluorescent C-dots–Fe_3_O_4_ nanoparticles are capable of separating MRSA and *Salmonella* DT104 superbugs from whole blood samples. This article also reports the strategy of antimicrobial peptide-conjugated fluorescent magneto-C-dots for efficient separation, identification, and complete disinfection of MDR superbugs from infected blood.

#### 4.1.7 Shape modification of MFQDs for improved applications

Magnetic quantum dots are immensely nurtured in diluted magnetic support for exploiting them into spintronics.^131^ Such materials show room temperature ferromagnetism after desired amount of doping.^132^ The most common is the core–shell shape where the core serves the magnetic character whereas the outside are providing optical feature like QDs. Wang *et al.* reported superparamagnetic (maghemite) core based CdSe/ZnS QDs conjugate for distinguishing cells and separation.^106^ Kim and group proposed a facile one-pot reaction where they prepared MFQDs composed of Co, magnetic metal core and CdSe semiconductor shell.^133^ These nanomaterials preserved the magnetic and optical characteristics of the component part approving potential applications in bioassays. A very interesting microwave assisted synthesis procedure of Fe_3_O_4_–CdSe core–shell nanocomposite was proposed by Zedan and coworkers.^134^ Magnetite Fe_3_O_4_ nanoparticles were applied as seeds for the purpose of heterogeneous nucleation and growth of the CdSe nano-shells. These MFNPs of 13% QY were almost monodisperse with an average size of 10 nm. These core–shell heterostructures delivered both fluorescence and magnetic features which is beneficial for simultaneous monitoring and separation in biomedical field. By regulating the microwave irradiation time, the optical properties of these MFNPs could be tuned which also can be useful to tune the shell (luminescent) thickness. Yi *et al.* also described surface conjugation of carbon quantum dots (CQDs) with cyclic DTPA dianhydride and further Gd^3+^ chelation for dual modal imaging probe.^135^ In a report magnetic-fluorescent nanobeads prepared by destabilization of a mixture of MNPs, QDs, and amphiphilic polymer followed by functionalization with folic acid was reported.^80^ By using acetonitrile or water as destabilizing agent, the dissemination of the NPs within the beads was regulated. Using acetonitrile resulted in the cluster formation of the MNPs in the center of the bead whereas the QDs were uniformly distributed. On the other hand, when selecting water, both QDs and MNPs were found to be uniformly distributed all over the bead. These different geometries leads to different magnetic responses of the beads. The former responded faster to an external magnetic field applied in comparison to water-destabilized nanobeads. The acetonitrile-destabilized nanobeads were applied for highly specific cell sorting study that showed recovering of targeted cells even at low percentage (up to 1%). Hsu *et al.* reported preparation of SiO_2_ nanohybrids comprising CuInS_2_/ZnS QDs and MNPs inside the W/O microemulsion system followed by the introduction of the functional groups at the surface of the SiO_2_ by condensation of TEOS and organosilane.^136^ The PEG functionality offered enhanced biocompatibility whereas the amine groups at the surface played the role to form amide linkage with the drug to achieve Pt(iv)-conjugated SiO_2_ nanohybrids. With respect to free Pt(iv) anticancer drug, the Pt(iv)-conjugated SiO_2_ nanohybrids exhibited higher cytotoxicity suggesting the potential of using SiO_2_ nanohybrids. Also these nanohybrids unveiled promising results for dual-modality imaging probes for cancer diagnosis and chemotherapy. Different types of MFQDs developed for various biomedical applications in the last few years are tabulated in Table 1.

**Table 1:** Summary of bi-functional MFQDs developed for different biomedical applications in the last few years

Types	Components	Sizes (nm)	Applications	Magnetic property	Ref.
Core–shell	CuInS_2_–Zn_1−*x*_Mn_*x*_S	2.5–4	MR and fluorescence imaging of cancer cells	*T*_1_ relaxivity (*r*_1_) (mM^−1^ s^−1^), ∼7.2	67
Mn doped QDs	Zn–Cu–In–(S,Se)/Zn_1−*x*_Mn_*x*_S	8 ± 2	*In vivo* imaging of the regional lymph nodes in mice in both NIRFI and MRI	*T*_1_-relaxivities up to 1400 mM^−1^ [QD] s^−1^ at 7 T and 300 K	68
Gd-based CuInS_2_/ZnS QDs	QD@PMO–Gd–FA	∼2.5	Dual modality magnetic resonance/optical imaging	*T*_1_ relaxivity (*r*_1_ = 3.7231 mM^−1^ s^−1^)	137
Engineered Gd-doped CQDs	Gd-doped CDs	∼18	MR imaging-guided radiotherapy: *in vivo* evaluation of radiosensitivity in mice bearing herps tumors	*r*_1_ relaxivity 6.45 mM^−1^ s^−1^	127
Silica nanohybrids integrated system	SiO_2_ nanohybrids containing CuInS_2_/ZnS QDs and Fe_3_O_4_	15–60	Cisplatin anticancer drug delivery into tumor cells for suppressing the growth of MCF-7 breast cancer cells	*T*_1_ relaxivity (*r*_1_ = 214 mM^−1^ s^−1^)	136
Nitrogen, cobalt co-doped CQDs	N,Co-CDs	2–4.8	Detection of cholesterol and uric acid	Saturated magnetization value of 1.476 emu g^−1^ and a coercive force (*H*_c_) of 9.68 × 103 Oe	128
Core–shell	Folic acid-conjugated ZnS : Mn/ZnS QDs	5.2 ± 1.0	Confocal microscopy with biphotonic excitation of T47D cancer cells	—	138
Ternary hollow nanospheres	Cd_0.57_Mn_0.43_S Cd_0.29_Mn_0.71_S	200–300	*In vivo T*_1_-weighted transverse MR images of mouse liver	*r*_1_ value 6.8 & 4.3 mM^−1^ s^−1^	139
Transition metal ion doped CQDs	Mn^2+^, Fe^2^+, Co^2+^, and Ni^2+^ doped C-dots	3.12, 2.73, 3.69, 2.72	*In vivo* magneto-fluorescent dual modality bioimaging of zebrafish	*r*_1_ relaxivity: for Mn/C-dots & Ni/C-dots 0.341 and 0.356 mM^−1^ s^−1^, respectively	140
Core–shell	Fe_3_O_4_–CdSe	∼10	Nanosensing, molecular separation, and bio-assaying	Superparamagnetic at 300 K and become ferromagnetic at 5 K	111
Gd(iii) chelates functionalized CQDs	CQD–DTPA–Gd	4–6	Fluorescence and magnetic resonance dual-modal bioimaging	Longitudinal relaxivity 56.72 mM^−1^ s^−1^	135
Paramagnetic GQDs	Folate–GdGQDs	1.2	Dual-modality bioimaging and tumor-targeted drug delivery	Relaxivity *r*_1_ 11.49 mM^−1^ s^−1^	141
Graphene oxide QDs decorated magnetic nanoplatform	GOQDs-coated amine functionalized MNPs	∼40	Efficient capture & two-photon imaging of rare tumor cells	Saturation magnetization of 37.8 emu g^−1^	142

## 5. Magneto-fluorescent heterocrystals

**Fig. 11:**
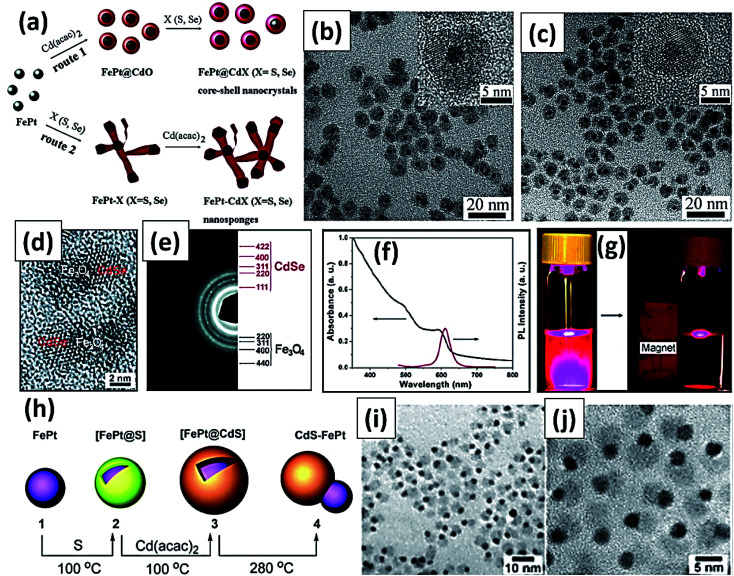
(a) Synthetic scheme of CdS decorated FePt heterocrystalline nanoparticle (b) TEM image of FePt@CdS core–shell nanocrystals (inset: magnified image of HRTEM image) (c) TEM image of FePt@CdSe core–shell nanocrystals (inset: magnified image of HRTEM image). Reproduced from ref. 105 with permission from American Chemical Society, copyright 2007. (d) HRTEM image and (e) SAED pattern of Fe_3_O_4_–CdSe heterodimers (f) the UV-visible and fluorescence spectra of Fe_3_O_4_–CdSe nanoparticles in hexane solution (g) fluorescence images of Fe_3_O_4_–CdSe nanoparticles in hexane solution before and after attraction by a small magnet (excited at 365 nm). Reproduced with permission from ref. 148 with permission from American Chemical Society, copyright 2008. (h) Schematic illustration for synthesis of CdS/FePt core–shell magnetic quantum dots and formation of heterodimer (i) TEM image of heterodimer (j) magnified HRTEM image of heterodimer showing surface attachment of quantum dots. Reproduced with permission from ref. 103 with permission from American Chemical Society, copyright 2004.

Heterocrystals are a special class of materials with two/more distinct materials. These materials could be in a core–shell morphology or in an asymmetric phase separated by heterodimer architecture. The most common magneto-fluorescent heterocrystals are superparamagnetic nanocrystals prepared *via* high temperature decomposition method. The core–shell heterocrystals reported by several scientists are Co@CdSe,^133^ FePt@Cd(S,Se,Te)^105,143^ and FePt@Pb(S,Se).^144^ The magneto-fluorescent heterodimers reported elsewhere are FePt–(Cd,Zn,Pb)S,^103,145^ FePt–Pb(S,Se),^144^ γ-Fe_2_O_3_–(Zn,Cd,Hg)S,^146^ γ-Fe_2_O_3_–CdSe^147^ and Fe_3_O_4_–Cd(S,Se).^148^ The divergences in the structures of the magnetic heterocrystal quantum dots come from the mismatching in the lattice array between magnetic components and the quantum dots. Nonetheless, these structural anomalies result from variations in the synthetic parameters such as temperature during reaction, sequential addition of precursor materials, and surface promotion agents. In a report, Goa *et al.* illustrated nanostructures containing core of FePt MNPs and semiconducting chalcogenides as the shell through a series of reactions in a one-pot method (Fig. 11a).^105^ HRTEM images revealed some degree of aggregation and fusion for the FePt@CdS nanocrystals (Fig. 11b) while the FePt@CdSe core–shell nanocrystals (Fig. 11c) showed better monodispersity. The HRTEM images indicated that both FePt parts and CdS or CdSe parts were crystalline in nature. Later these authors also reported the intracellular manipulation of fluorescent MNPs by magnetic force.^148^ These particles possess superparamagnetism, that permit their movements in the intracellular system to be governed by magnetic force and tracking using fluorescent microscope. The HRTEM (Fig. 11d) image suggested that the darker region and lighter ones were corresponded to Fe_3_O_4_ and CdSe in the heterodimer, respectively. Fig. 11e showed the EDP pattern of the Fe_3_O_4_–CdSe nanoparticles. The UV-visible absorption peak ∼600 nm (Fig. 11f) accredited to the absorption of the CdSe part and the emission peak of the nanoparticles ∼610 nm was observed. Fe_3_O_4_–CdSe nanoparticles in hexane solution was visualized under UV before and after introduction of a small magnet (Fig. 11g). In a report, Gu and coworkers reported bifunctional heterodimers of CdS and FePt by sequential incorporation of sulfur and Cd(acac)_2_ into the FePt colloid solution (Fig. 11h).^103^Fig. 11i and j displayed the TEM and HRTEM image of heterodimers CdS–FePt. The author proposed that this simple technique may lead to large scale production of various heterostructures. Bimetal doped (Fe, Pt) magnetic quantum dots (Cd(S, Se)) were synthesized at 260 °C that resulted in core–shell nanoparticles, whereas for identical precursor systems but under different condition (higher temperature), the outcome is a heterodimer. These heterodimer is an effect of high temperature internal sintering/coalescence among the metal–semiconductor domains.^144^ Heterodimers are significant for dual surface behavior especially designed for multimodal probes. In general for targeted specific activity with high precision, heterodimers are engineered by surface ‘grafting to’ approaches. Some of the researches follow dual attachment of drug molecules and target agents both in one particulate nano-heterodimer. The dual characteristics of these particles are on demand to mitigate the limitations of fluorophore-drug dual binding efficacy in one particle. These also solve the tedious synthetic approaches towards multimodal probe fabrications. But due to di-variant nature of the domains, heterodimers are always suffering from thermodynamic stability and entropy related factors in their interfaces. Moreover, the quantum yields of these particles are also hampered due to limited attachment of the fluorophores. The PL quenching is also observed due to improper matching of the crystal domains, interfacial doping, restrictive electron passage through the interfaces, and band edge neutralization of the fluorophore.^133,149^ The superparamagnetic character of the magnetic nanodots is comparatively easier to retain during their fabrication. But, interfacial perturbation due to mismatching of domains sometimes affects the quality of the magnetic behavior.

## 6. Magnetic and luminescence bifunctionalized Janus particles

**Fig. 12:**
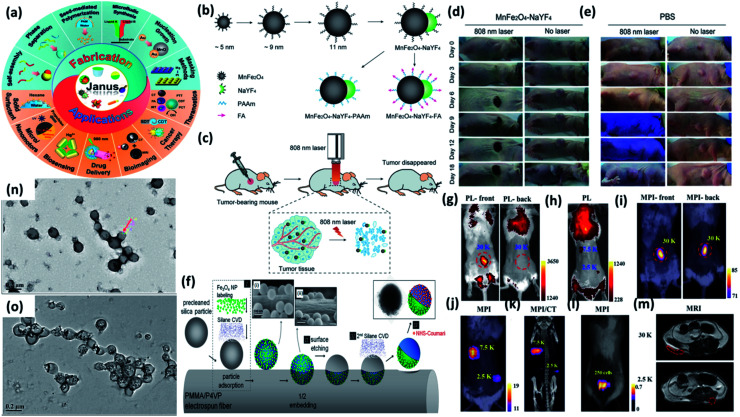
(a) Janus particles: fabrication and applications. The inner circle represented different Janus structures. Reproduced from ref. 157 with permission from American Chemical Society, copyright 2021. (b) Synthesis and modification procedure of MnFe_2_O_4_–NaYF_4_ Janus NPs (c) application in tumor photothermal therapy upon 808 nm laser irradiation. *In vivo* photothermal therapy: tumor bearing mice intratumorally injected with (d) MnFe_2_O_4_–NaYF_4_ and (e) PBS and exposed to 880 nm laser irradiation. Reproduced from ref. 158 with permission from John Wiley and Sons, copyright 2017. (f) Fabrication technique of Fe_3_O_4_/coumarin Janus particles. Reproduced from ref. 159 with permission from American Chemical Society, copyright 2014. (g and h) Front or back view of fluorescence imaging of a mouse after local subcutaneous injection of Fe_3_O_4_@PFODBT-COOH labeled cells (i and j) front view or back view of 2D projection MPI of mouse after injection of labeled cells. (k) 3D MPI and CT imaging of mouse after local subcutaneous injection of labeled cells. (l) Overlay of white light picture and 2-D projection MPI image. (m) MRI transverse images of mouse body after local subcutaneous injection of cells labeled with Fe_3_O_4_@PFODBT-COOH. Reproduced from ref. 160 with permission from American Chemical Society, copyright 2018. TEM images of (n) PMCP-10 and (o) PMCP-30. Reproduced from ref. 161 with permission from Multidisciplinary Digital Publishing Institute, copyright 2019.

In 1991, during the Nobel Laureate Lecture entitled Soft Matter of Pierre-Gilles de Gennes, he proposed the concept of ‘Janus particles’ as anisotropic nanomaterials to the scientific community. His research group reported an amphiphilic glass bead with two hemispheres of polarity and nonpolarity and since then Janus particles (JPs) have attracted attention owing to their interesting physical and chemical features.^150,151^ Janus nanoparticles (JNPs) have broken the traditional symmetry by not only assimilating different components into one structure but also supporting various functionalizations with combined structure.^152,153^ Different synthesis method and biomedical applications of JNPs was presented in Fig. 12a. In the past few years, the use of versatile magnetic-plasmonic, magnetic-luminescent JNPs has witnessed immense interest as contrast agents for multimodal imaging, and cancer therapy.^154–157^

### 6.1 Development of bifunctionalized JPs and their therapeutic uses

In this section, we will discuss the development of JPs comprising of magnetic and luminescent features in the past few years. The assimilation of magnetic and luminescence property into one structure of JNPs make them applicable more towards MRI imaging, magnetic-driven drug delivery, and cancer therapy as compared to micro/nanosphere or core/shell nanoparticles which generally showed single functionality. Although there has been notable progress in the advancement of magnetic-luminescent nanostructures for cancer therapy during past few years, the integration of photothermal and upconversion luminescence features in a single entity for cell labeling and tumor photothermal therapy has not been explored until Wu *et al.* developed dumbbell-like MnFe_2_O_4_–NaYF_4_ JNPs.^158^ The dumbbell-like MnFe_2_O_4_–NaYF_4_ JNPs were prepared (Fig. 12b) by a two-step thermolysis approach using Mn(acac)_2_ and Fe(acac)_3_ as precursors. After that, the dumbbell structure was formed through epitaxial growth of lanthanide cation (Yb or Er) doped NaYF_4_ on the MnFe_2_O_4_ nanoparticles. These MnFe_2_O_4_–NaYF_4_ JNPs were applied for photothermal therapy for killing cancer cells in *in vivo* as shown in Fig. 12c. As shown in Fig. 12d and e when tumor-bearing mice (*n* = 6) were intratumorally treated with MnFe_2_O_4_–NaYF_4_ JNPs, substantial suppression in tumor growths was observed after 808 nm laser irradiation. The tumors became like black spots post treatment and completely disappeared after 18 days of irradiation. Chao *et al.* reported on silica cored JNPs with two hemispheric surfaces individually functionalized (Fig. 12f) with Fe_3_O_4_ nanoparticles and coumarin-466.^159^ These JNPs were further examined on the basis of their anisotropic fluorescent emission along with their magnetically induced orientation. Rao *et al.* developed encapsulated Fe_3_O_4_ nanoparticles in fluorescent semiconducting polymers to obtain Janus Fe_3_O_4_@semiconducting polymer nanoparticles afforded efficient cell labeling and sensitive magnetic particle imaging (MPI) monitoring after implantation into mice.^160^ The author discussed that their synthesized Fe_3_O_4_ JNPs showed three times and seven times that of the MPI signal of commercial MPI tracer (Vivotrax) and MRI contrast agent (Feraheme), respectively, at the same concentration of Fe. To compare the imaging contrast and depth attenuation of three imaging modalities *in vivo* Fe_3_O_4_@PFODBT-COOH labeled HeLa cells implanted into mouse whole body. Fig. 12g–m showed fluorescence, MPI, and MRI of Fe_3_O_4_@PFODBT-COOH labeled HeLa cells implanted mouse body. Li and coworkers developed a simple method to synthesize JPs comprised of fluorescent polyurethane and hydrophobic nano Fe_3_O_4_ by a method of mini-emulsification and self-assembly.^161^ The nanostructures of the PMCP (PU-MHHNA magnetic composite nanoparticle) with various HMNP content were studied using TEM analysis. In PMCP-10 (Fig. 12n), most of the HMNPs were encapsulated but they were not uniformly dispersed owing to the phase separation. With increasing HMNP content to 30%, most of the nanoparticles were fully occupied with HMNPs as shown in Fig. 12o causing a lower ratio of JPs. The JNPs featured unique properties, excellent dispersity, storage stability, and biocompatibility which is advantageous for their application in biomedical areas.

## 7. Magneto-fluorescent QDs in therapeutic applications

The breakthroughs in cancer research have been gained through some high valued synergistic therapeutic controls where chemotherapy and other diagnostics mingled together. In this context, researchers in materials engineering domain inferred that proper tracking of nanoparticles by using different diagnostic tools becoming significant in next generation medical marvels. Fluorescence and magnetism have been amalgamated to induce a special class of fluorescent guided magnetic nanoparticles (MNPs). Iron oxides nanoparticles (IONPs) and surface carbon decorated magnetic nanoparticles have been drawing attention for their *in vivo* medical treatment. These materials can penetrate through cell membrane and response in a wide frequency range of alternating magnetic field. These nanoparticles could be tracked, imaged, and remotely controlled non-invasively. Magnetism is related to their domain size distribution. Normally, if the size of the magnetic nanoparticles is going down especially below 10 nm, the particles turn into superparamagnetic. Superparamagnetic nanoparticles are transient tiny magnets showing fast and immediate magnetism against external magnetic field and fast demagnetization when the external field is removed. Small particles are also superior compare to larger sized nanoparticles regarding their biodegradation behavior.

**Fig. 13:**
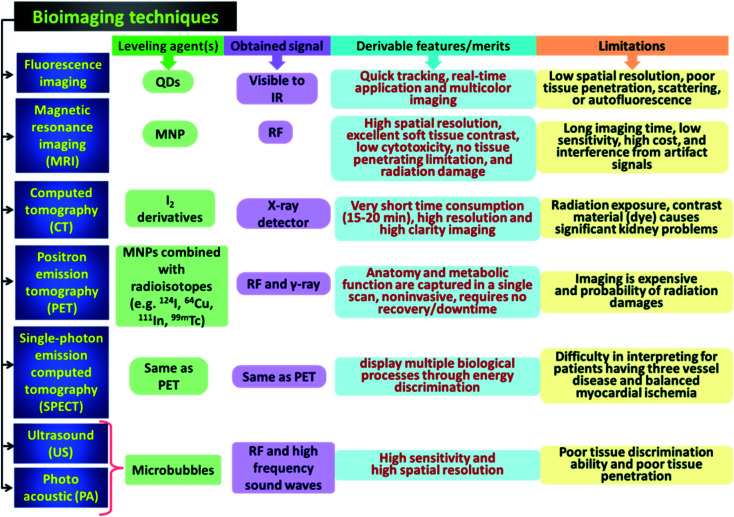
Brief classification of various imaging techniques for marking cells/tissues and their advantages-disadvantages.

When quantum dots (QDs) meet MNPs, the resultant product is a marvel of pure blending between excellent optical property (from QDs) and magnetism (from MNPs). Combined properties of QDs and MNP have applicability in cancer research especially in tumor cell distinguishing, dynamic real time tracking, fluorescence images, drug delivery, magnetic resonance (MR) imaging and so on. A brief classification of different bioimaging techniques for cells/tissues monitoring and their merits and limitations were presented in Fig. 13.

## 8. Fluorescent bioimaging probes

**Fig. 14:**
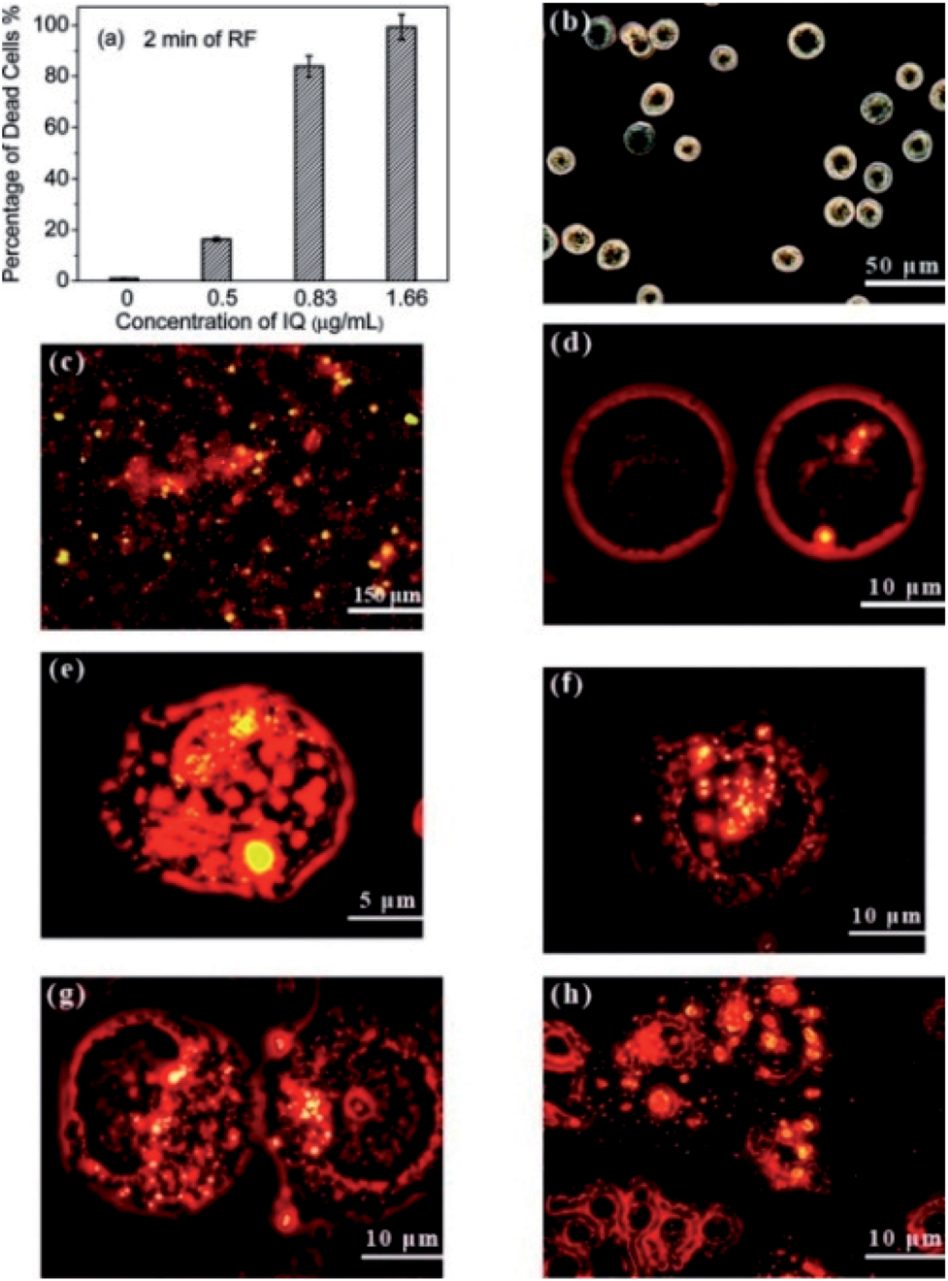
(a) Percentage of dead cells as the function of IQ concentrations under the RF treatment of 2 min. Confocal microscopic images of (b) normal Panc-1 cells, (c) IQ nanocomposite incubated with Panc-1 cells for 24 h. (d) Nanocomposites (without and with UV radiation) were uptaken into the cell cytoplasm, and they were found to agglomerate around the nuclear membrane, and a small number of them crossed the nuclear membrane into the nucleus. (e–h) Cell shrinkage, membrane blebbing, the disintegration of the nuclear membranes, and apoptotic bodies as they resulted from the RF heating process. Reproduced from ref. 5 with permission from American Chemical Society, copyright 2010.

Bioimaging is a leveling strategy of cells to visualize or sense though specific devices. The machines used for such biomedical imaging methods are versatile depending on their mode of activities. The diagnostic tools in this context are computed tomography (CT), magnetic resonance imaging (MRI), optical fluorescence (FL) imaging, positron emission tomography (PET), single-photon emission computed tomography (SPECT), ultrasound (US) imaging, photo acoustic (PA) imaging, and so on.^162^ Among these diagnostic tools FL imaging and MRI are most widely used in medical area due to their totally non-invasive technique of application and there are no usages of harmful radiation. QDs are better compare to the commercially available fluorescent dyes/pigments due to possessing high photostability, large stokes shifts, and broad excitation spectra alleviating QDs to perform in *in vivo* applications. Gao *et al.* carried out an excellent experiment that could prove clear dissimilarities between cells. They tagged one cell line with green fluorescent protein (GFP) and another one with QDs. They showed that for *in vivo* application, QDs marked cells were clearly distinguishable whereas GFP tagged cells were not visualized. Moreover, they also performed multicolor cell imaging with a single light source. Near infrared (NIR) QDs leveling also is under high demand in cancer diagnostics because of its higher penetration and in depth visibility for tissues. NIR emits comparatively less autofluorescence but it is worth mentioning that some toxic elements like (Cd, Pb) derived QDs are restricted in this context. Wen *et al.* derived Ag based sulphide and selenides for NIR emitting QDs for *in vivo* application.^163^ Corato *et al.* developed multifunctional nanobead from manganese iron oxide decorated with inorganic QDs for sorting of cancer cell.^80^ They fabricated an alternating copolymer made of poly(maleic anhydride-*alt*-1-octadecene) filled with manganese iron oxide core–shell CdSe/ZnS fluorescent nanoparticles with almost narrow size distribution. They also functionalized these nanobeads with folic acid promote its receptor like behaviour towards cancer cells. They tagged the cancer cells followed by application of 0.3 Tesla external static magnetic field to separate out from the mixture. Surprisingly all those tagged cells were separated within one hour span from the starting. Electrochemiluminescence (ECL) was attached as a surplus feature of fluorescent magnetic nanoparticles for fabricating gold nanoparticles that could be used for cancer cell assay.^164^ Cancer diagnostics and curing were simultaneously carried out using silica coated iron oxide functionalised QDs.^5^ They incubated the cells tagged with fluorescent magnetic QDs followed by exposure to radio frequency (RF) for several minutes. They showed that cell apoptosis was observed with increasing the magnetic nanoparticles content inside cells (Fig. 14). As showed in the images the RF exposed cells were gradually dead after showing membrane shrinkage, and nuclear disintegration.

Sun *et al.* developed CdTe surface decorated magnetite nanoparticles by covalent linkage followed by thiol capping with thioglycolic acid.^2^ Thioglycolic acid acts as receptor of HeLa cells. In this work they conjugated the iron oxide to the antibody of the cell and observed under FL microscope. These kinds of nanoparticles could be very significant for multimodal images. Ma *et al.* magnetic quantum dots with NIR emission by reverse microemulsion method.^165^ They conjugated antibody to the nanoparticles and used this as human breast cancer probe. γ-Fe_2_O_3_/CdSe QDs were prepared in presence of surfactant followed by binding them with antibody. These magnetic nanoparticles were tested against breast cancer cells (KPL-4) and confirmed by FL microscopy. Liu *et al.* developed sensitive electrochemiluminescence (ECL) immunosensor by graphene/iron oxide composite decorated with CdTe QDs for the detection of prostate specific antigen (PSA).^166^ Here graphene/iron oxide played the role of immunosensing probe whereas inorganic QDs (CdTe) acted as signal amplifier. They deflected very small quantity of PSA by this technique and the range of detection was in between 0.003–50 ng mL^−1^. Yu *et al.* developed superparamagnetic beads for detection of cancer biomarkers in microfluidic chips with a very low detection limit of 3.5 ng mL^−1^.^167^ Iron–biopolymer complex were used as magnetofluorescent nano-vehicle for drugs, fluorescent probing of cancer cells and targeted killing of cancer cells.^168^ This work reported single step formation of QDs conjugated magnetic nanoparticles and grafting of them with photosensitizer (riboflavin). These nanoparticles also showed targeted precise killing of cancer cells by release of doxorubicin drug molecules. Micrometastases of lung cancer were detected by using MNPs–QDs conjugate where the nanoparticles were bound to antibody and targeted to cancer cells.^169^ Chen *et al.* developed CNTs lumen decoration by magnetite (Fe_3_O_4_) and outer surface decoration with transferrin and QDs. This assembly have several significances like prevention of magnetic agglomeration, high drug loading capability, biocompatibility, and exposed receptor for easy cancer cell targeting.^170^ Transferrin is a receptor protein that is overexpressed on HeLa cells. Besides these, the work also showed how the drug delivery was monitored by magneto-fluorescent probes. There are several researchers also worked on surface decorated iron oxides for cell penetration activity and imaging. Most researchers used semiconductor QDs for better FL intensity. Aptamer conjugated Fe_3_O_4_@MoS_2_ nanosheets were synthesized for stable magnetic nanoparticles that were used for cell sorting experiment and detection of tumor cells.^171^ Anionic polymers are on high demand for stabilizing fluorescent magnetic nanoparticles. In this context, poly(styrene sulfonate) (PSS) was used as surface coating agent for magnetic carbon nanotubes (CNTs). These nanoparticles were used as drug carrier and biolebeling of cancer cells.^170^ Zhang *et al.* developed Gd-doped CdTe conjugated nanoparticles that performed bimodally as FL labeling agent as well as MRI contrast agent. For precise cancer cell targeting they modified those nanoparticles with folic acid (FA) on to their surfaces.^4^ There are several works have been performed by fluorescent quantum dots tagged magnetic nanoparticles for cell tracking and precise delivery of cancer drugs as shown in Table 2.

**Table 2:** Surface decorated QDs-conjugated magnetic nanoparticles and their applications in the last few years

Magnetic component	QDs	Surface decoration	Applications	Ref.
Manganese iron oxide	CdSe–ZnS	Poly(maleic anhydride-*alt*-1-octadecene)	Cancer cell targeting and sorting	80
Fe_3_O_4_@MoS_2_ nanosheets	GQDs	Aptamer [epithelial cell adhesion molecule (EpCAM) receptors]	Sensitive separation and detection of circulating tumour cells	171
Fe_3_O_4_-filled carbon nanotubes (CNTs)	SiO_2_ coated CdTe	PSS	Cancer-targeted imaging and magnetically guided drug delivery	170
Fe_3_O_4_/SiO_2_	CdSe/ZnS	—	Multimodality imaging of breast cancer tumours	165
Fe_3_O_4_	CdTe	CTAB	Imaging colon carcinoma cells	172
Fe_3_O_4_	CdTe	Mucin 1 protein aptamer	Bio-labelling and signal amplification	173
Fe_3_O_4_	CdS/ZnS	Goat anti-rabbit IgG antibody	Cancer biomarkers for serum	174
Gd-doped CdTe	Gd-doped CdTe	Folic acid	Tumour-targeted FL/MR dual-modal imaging	4
Fe_3_O_4_	GOQD	1,6-Hexadiamine	Glypican-3 (GPC3)-expressed Hep G2 liver cancer tumour CTCs from infected blood	142
SiO_2_@Fe_3_O_4_	CdTe	Thioglycolic acid	Immuno-labelling and fluorescent imaging of cancer cells	2
Streptavidin-functionalized magnetic bead	QD630	Octylamine	Quantification of circulating tumour cells	175
Bacterial magnetic particles	Streptavidin-QD	Fetal bovine serum (FBS)	Magnetic separation and fluorescent labeling of cancer cells	176
FeN	Chitosan CDs	Riboflavin and folic acid	Chemotherapy and effective cancer treating	168
Streptavidin@iron oxide	QD605	Aptamer LY-1	Recognition and capture of metastatic hepatocellular carcinoma cells	177
Gd	CuInS_2_/ZnS	SiO_2_	Fluorescence imaging of cancer cells (human pancreatic cancer cell line BXPC-3)	178
Zn_1−*x*_Mn_*x*_S	CuInS_2_	CTAB	*T*_1_ contrast agent and fluorescence imaging of cancer cells	67
Gd	CdSe/ZnS	BSA	Imaging of tumour angiogenesis	179
γ-Fe_2_O_3_	CdSe/ZnS	Poly(styrene/acrylamide)	Detecting and isolating multiple types of tumour cells	180
Gd–Zn–Cu–In–S	Gd–Zn–Cu–In–S	ZnS	MR and FL imaging	181
Gd	C-dots (citric acid)	Apoferritin	Bioimaging and targeted therapy	182
Fe_3_O_4_	Porous silicon	ω-Alkene	FL/MR bimodal imaging of tumour *in vivo*	183
CuInS_2_	ZnS : Mn	Dihydrolipoic acid-poly(ethylene glycol) (DHLA-PEG)	Fluorescence/MR dual-modality probe	53
Streptavidin-coated Fe_3_O_4_	CdSe	—	Immunofluorescent assays	184
Gd^3+^ doped Y_2_O_3_	Eu^3+^ doping	Folic acid	Bi-modal fluorescence and magnetic imaging of cancer cells (A549)	185
SPIONs	CdSe/ZnS	Cilengitide	Dual-imaging guiding cancer surgery	186
Fe_3_O_4_	CdSe/ZnS	Cyclic Asn-Gly-Arg	Imaging of tumor angiogenesis	187
Magnetic beads labeled with anti-human CD4	Quantum dot-625 streptavidin	—	Immunomagnetic separation at low concentrations	188
Magnetic beads	QD-525, QD-585, and QD-625	BSA	Multiplexed fluoroimmunoassay of lung cancer biomarkers	189
Mn-doped ZnS	Mn-doped ZnS	—	Dual-modal bio-imaging	190
Fe_3_O_4_	CdTe	Silica	Human embryonic kidney 293 cells (HEK293) lebelling	191
Fe_3_O_4_	ZnS : Mn^2+^	Poly(NIPAAm-*co*-AAm)	Temperature triggered drug release, bioimaging and *in vivo* tumor inhibition	192
Fe_3_O_4_	CdTe	Peptide	*In vivo* targeted imaging and hyperthermia therapy of prostate cancer	193
Gd–N,S-doped QDs	Gd–N,S-doped QDs	Folic acid	Imaging and chemotherapy	194
γ-Fe_2_O_3_	CdSe/ZnS	Dimercapto-succinimid acid (DMSA)	Cell separation	106

## 9. Magnetic resonance imaging (MRI)

**Fig. 15:**
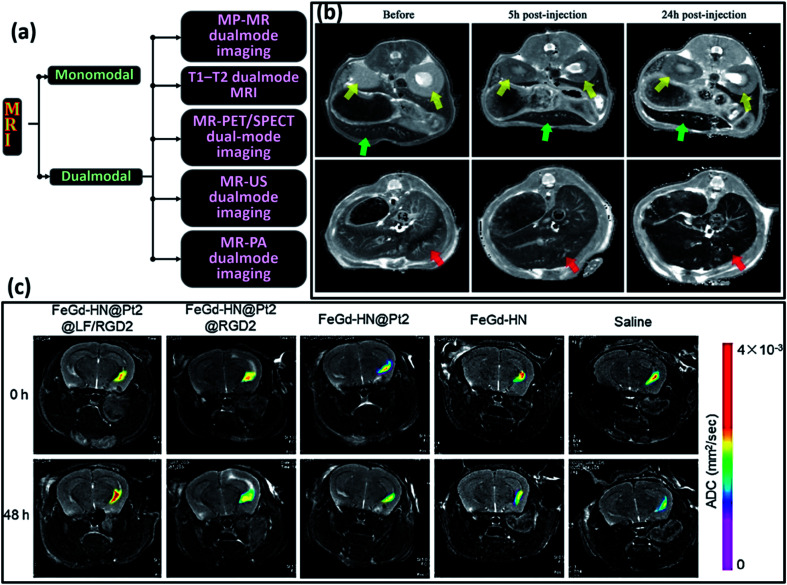
(a) Classification of different MRI techniques (b) *in vivo* MRI *T*_2_ maps before administration of a 9% dose of contrast agent (NE-SPIONs 4 h) and 5 and 24 h after injection. Each image shows axial sections through the abdominal region of mice. Kidneys, spleen, and liver are, respectively, indicated by yellow, green, and red arrows. Reproduced from ref. 195 with permission from American Chemical Society, copyright 2019. (c) Therapeutic efficacy of ferroptosis therapy (FT) in orthotopic brain-tumor-bearing mice measured by diffusion weighted MRI. Apparent diffusion coefficient (ADC) parametric maps of the orthotopic brain-tumor-bearing mice before or after treatment with FeGd-HN@Pt2@LF/RGD2, FeGd-HN@Pt2@RGD2, FeGd-HN@Pt2, FeGd-HN, or saline. Reproduced from ref. 199 with permission from American Chemical Society, copyright 2018.

Magnetic resonance imaging or MRI has superior imaging technique compared to other imaging techniques due to its spatial resolution, high soft tissue contrast, and non-usage of radioactive elements. MRI can be classified into two major categories: monomodal and dualmodal. When the dualmodal MRI techniques are combined with other imaging techniques that can be classified in several sub-groups as shown in Fig. 15a. Though it has plenty of advantages, but it has limitations to describe clearly the detailed anatomical activity (Fig. 15b and c). Wallyn *et al.* studied MR *in vivo* imaging and the quantification of the contrast agent buildup.^195^ After administration of the product to the blood, the muscles were selected as the regions of interest to identify and quantify the hyposignal because of the contrast agent circulation in the blood. Other regions of interest were selected (Fig. 15b) in the liver, spleen, and kidneys and the corresponding signal drop was quantified by *T*_2_- and 
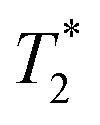
-weighted MRI. Thus paramagnetic nanoparticles are of good interest for MRI contrast agent. Gd based contrast agents are of wide use but small amount of Gd couldn't perfume well as contrast agent. Hence, iron oxides are more significant for this context. Iron oxides are highly abundant material showing excellent biocompatibility, prone to surface engineering, and size controllable magnetic quality. As per the clinicians, 100 mg iron oxides per kg of body weight do not show any detrimental effect on human body, even 600 mg of iron oxide also has been reported to be administered.^196^ Iron oxides inside body are captured by macrophages in reticuloendothelial system (RES) followed by eradication/degradation in lysozymes. In case of MRI, magnetic nanoparticles adjacent water molecules are affected and their spin–spin relaxation time (*T*_2_) has been decreased. This results show increasing of the brightness and good contrast. This *T*_2_ is inversely related to the relaxivity (*R*_2_). Thus it can be uttered if the relaxivity has high values, contrast will, be more. Size of nanoparticles is another contributing factor for getting good contrast. For bulk materials all dipole spins are in parallel orientation. But in nanodimension the spins are perturbed and a spin-glass like disordered state is generated. This spin-glass formation in the surfaces of nanoparticles affects their inherent saturation magnetization. The magnetizations of different size magnetite particles are 25, 43, 80, and 101 emu g^−1^ for the particles having diameter of 4 nm, 6 nm, 9 nm, and 12 nm respectively.^197^ The surface spin-glass effect becomes more pronounced when the size of the nanoparticles were decreased. This dominance of surface defects is noticed because their magnetic moments are decreased. When the magnetic moments are increased, the *R*_2_ becomes dominant and as a result better contrast is obtained. Metal ferrites also show significant contrast for MRI. When Mn is used for metal ferrite, it shows comparatively better saturation magnetization due to high spins whereas the magnetic moments are decreasing when the manganese (Mn) is replaced to Fe, Co, and Ni. Thus, Mn-based ferrite is the best material for MRI contrast agent.^198^

## 10. Targeted MRI techniques

**Fig. 16:**
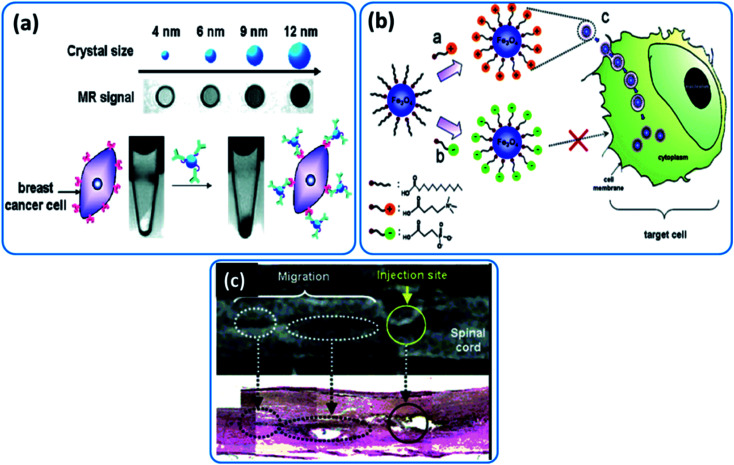
(a) *In vitro* MR detection of breast cancer cell lines by 2,3-dimercaptosuccinic acid (DMSA)-coated water-soluble Fe_3_O_4_. Reproduced from ref. 197 with permission from American Chemical Society, copyright 2005. (b) Graphical illustration of the ligand exchange procedure and utilization in cellular labelling by cationic MNPs (c) *in vivo* MR tracking and histological examination by using Prussian blue of cationic MNPs-labelled neural stem cells in a SD rat spinal cord. Reproduced from ref. 203 with permission from American Chemical Society, copyright 2005.

Surface decoration of magnetic nanoparticles can lead to the target specific diagnostics of cancer. The mostly used magnetic nanoparticle is iron oxides that have large amount of surface polarities and anchored to specific molecules/receptors for cancer cell targeting devices. As per the statistics, less than 15% of the total cancer patients are precisely diagnosed in the verge of stage I or II. Magnetic nanoparticles especially iron oxides have better contrast compared to other nanoparticles in MRI studies. Several successful examples are lies in the literatures.^200^ A group of researchers developed method for iron oxide based molecular imaging of breast cancer cells. Breast cancer cells are overexpressed to human epidermal growth factor receptor 2. Iron oxides with relaxivity coefficient (*r*_2_) of 218 mM^−1^ s^−1^ are prepared by tagging them to ‘receptor 2’ targeted antibody Herceptin that was successfully projected to breast cancer cells.^197^ In another work iron oxide-Herceptin conjugated material was used for *in vitro* cancer detection probe for Bx-PC-3 cell lines as shown in Fig. 16a. Dopant effect is also very much pronounced in MRI where manganese doped spinel (MnFe_2_O_4_) nanoparticles were used as magnetic probe for precise detection of cancers. MnFe_2_O_4_ has a high value of *r*_2_ compared to pure iron oxide (Fe_3_O_4_) that's why it showed better contrast than simple iron oxide.^198^ These nanoparticles were used in application of detecting tiny metastasis growth in mouse (∼50 mg). This manganese doped spinel also showed ∼12% greater value in *R*_2_ magnitude. Antibody conjugated magnetite is also a versatile area for detection of other typed cancer cells. Iron oxide conjugated rch24 antibody was used for colon cancer cells. Colon cancer cells have a specific antigen called carcinoma embryogenic antigen (CEA) that were targeted by rch24 conjugated iron oxide with a better contrast even in *in vivo* applications.^201^ Magnetic noble metal based nanoparticles are also drawing attention in this area. Fe–Pt–Au nanoparticles were conjugated with antibody named HmenB1 that are highly specific to neuroblastoma cells (CHP-134) and overexpressed with polysialic acid. These nanoparticles were showing better black contrast in MR images.^202^

## 11. Cell trafficking

The migration of cells is called ‘cell trafficking’ that can be oversaw by MRI using magnetic nanoparticles. This method is very useful for solving the cellular pathways and fate of the cells after delivery. Magnetic nanoparticles conjugated with various transfection agents are used for this purposes.^204,205^ Iron-cobalt alloy magnetic nanoparticles showed very high contrast having *r*_2_ value of 644 mM^−1^ s^−1^ was suitable for MRI.^206^ These imaging was also compared to the commercially available MNPs (Feridex) where these alloy nanoparticles showed better contrast compared to Feridex. Cell permeation of MNPs is a crucial step. For this purpose MNPs are surface decorated with ligands for easy permeation/penetration of MNPs inside cells. These ligands could be cationic or anionic in nature. (3-Carboxypropyl)trimethylammonium chloride, a cationic surface active agent was conjugated to neural stem cells showing better transfection efficiency compared to anionic surface active agents.^203^ Fast cellular transfection of nanoparticles are highly desired feature for *in vivo* cellular MRI of cell trafficking. This process is also used for monitoring of the neural stem cells in longitudinal migration. The graphical illustration and migration of MNPs are shown in Fig. 16b and c.

## 12. Magnetic hyperthermia (MH) and photothermal therapy (PTT)

Hyperthermia could be defined as abnormal elevation of body temperature by some external means. It is completely different form of heat than fever or heatstroke. In this context, if we recollect the great physicians of Greece Hippocrates and Kos at 460 and 372 BCE respectively, one statement has quite significance here. As per their aphorism hyperthermia was projected as follows; ‘whenever medicines are not a cure, iron (knife) heals; whenever, knife is not a cure, fire (heat) heals. But if heat could not cure, it is an inevitable cureless’ (quae medicamenta non sanat; ferrum sanat. quae ferrum non sanat; ignis sanat. Quae vero ignis non sanat; insanabilia reportari oportet). In the very early times, hyperthermia was used for curing inflammation, body pain, arthritis, muscular spasms, and cysts. But later, the hyperthermia has been widely used in medical area to cure cancer in a non-invasive approach. Commercially, hyperthermia is applied in multiple ways like whole body hyperthermia (WBHT), wire applicators-based hyperthermia (WAHT), and heating source insertion hyperthermia (HSIHT). The detailing of these strategies is tabulated for better understanding in Table 3.

**Table 3:** Different strategies, activity, and applications of the hyperthermia treatment

HT category	Activity	Devices	Result	Ref.
WBHT	Primary HT, used for treatment of metastatic growth, used by covering patient except head	Thermal hollow tubes, hot blankets, wax	• Used for those patients having severe ailments from tumour growth	207 and 208
			• Superficial overheating, heterogeneous heat distribution	
			• Surface burning may occur	
			• Thermal lesions, discomfort	
			• High temperature may effect on cardiac problem, intravascular coagulations, reduction in blood plasma level, and capillary leak syndrome	
WAHT	Non-invasive local therapy, heat wave transportation by applicator to targeted organ/tumour	High intensity focused ultrasound, radiofrequency, microwave	• Major problem in the area having bones	209–211
			• Non-uniform heat distribution	
			• Utilization of extra capacitive plate for radiofrequency tuning	
			• Uncontrolled heating in the fat dominated region	
			• High frequency microwave heats small mocules and fast wiring of temperature	
			• Microwave in better ompared to radiofrequency for heat dissipation	
HISHT	Heating source inserted, invasive method, discomfort during therapy	Microwave, radiofrequency, F_0_M seeds	• Better homogeneity than precious types	211 and 212
			• Radiofrequency creates problem due to charging effect followed by burning	
			• Thermo seeds (F_0_M) make eddy current in interstitial hyperthermia	
			• It could offer heat dissipation without current to affected cells	

**Fig. 17:**
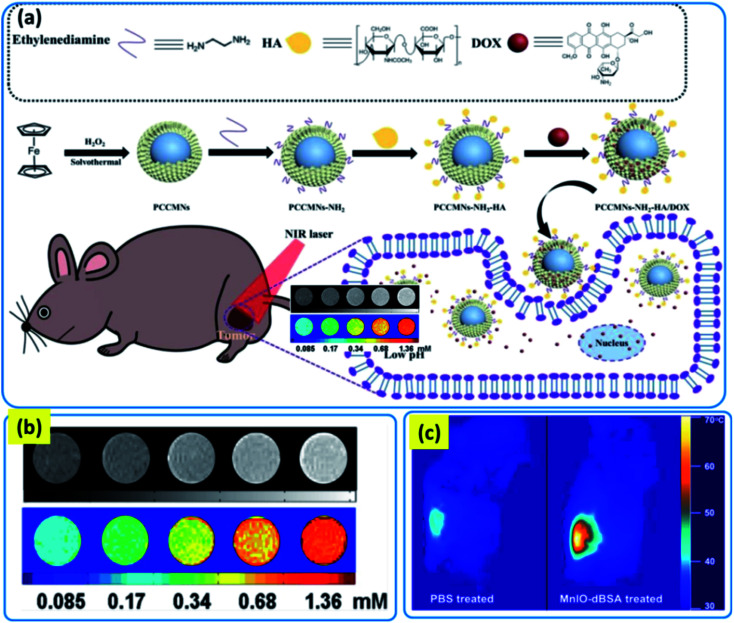
(a) Schematic description of the procedure of porous carbon nanoparticles coated magnetic nanoparticles preparation and synergistic PTT/chemotherapy of tumors. Reproduced from ref. 32 with permission from American Chemical Society, copyright 2019. (b) *T*_1_-weighted MR images (top) and their color-coded images (bottom) of the MnIO aqueous solution at various metal concentrations (Fe + Mn) (c) IR thermal images of PBS and MnIO-dBSA treated mice at the end of 5 min laser irradiation. Reproduced with the permission from ref. 221 with permission from American Chemical Society, copyright 2015.

Cancer diagnostics and treatment is highly inspired from the synergistic application of hyperthermia and photothermal therapy. These are localized therapeutic strategies by which specific domain tissue could be heated around 41–49 °C. This range is called hyperthermic range and beyond this temperature domain (50–59 °C) it is called thermoablatic range. Magnetic nanoparticles are used as the nanoheaters generating heat under oscillating/alternating magnetic field.^36^ Photothermal therapy (PTT) is little different in terms of compositional aspect. For PTT photoabsorber is required that coverts optical energy to thermal energy to kill cancer cells.^213^ The photoabsorbers are generally gold nanoparticles, carbon nanoparticles, and copper chalcogenides.^214^ As magnetic heating and PTT both own identical destination to kill cancer cells, these could be mingled together to achieve precise noninvasive therapy. Mesoporous silica based magnetic nanoparticles have much significance in hyperthermia therapy.^215^ Graphene quantum dots (GQDs) capped magnetic mesoporous silica nanospheres has been developed by a group of researchers showing dual modal therapy based on alternating magnetic field (AMF) and PTT. These nanoparticles were permeated to 4T1 human breast cancer cell lines and responded by external AMF and NIR laser. As a result localized heating phenomenon was observed followed by gradual destruction of cancer cells.^216^ Liao *et al.* reported WS_2_ quantum dots capped silica nanoparticles for PTT that was applied to kill colon cancer cells (HCT 116).^31^ Biocompatible superparamagnetic QDs are also playing an effective role on AMF heating as well as PTT. Justin *et al.* developed magnetite/GQDs based magneto-fluorescent nanoparticles for targeted delivery, precise control of release, and localized non-invasive cancer treatment.^217^ The additional profit for these nanoparticles is that they also showed MRI sensitivity. Wu *et al.* prepared ethylenediamine based QDs conjugated hyaluronic acid (HA) as drug carrier for NIR responsive drug delivery to kill cancer.^32^ Besides these they also claimed that these nanoparticles are *in vivo* MR guided. The synthesis and a brief working procedure have been given in Fig. 17a. Elbialy *et al.* developed magnetic gold nanoparticles for synergistic applicability of MH and PTT.^218^ They took magnetite as a magnetic core and coated with gold layers followed by binding it with a cancer drug; doxorubicin. They showed both *in vitro* as well as *in vivo* applicability of the nanoparticles for potential PTT and MRI contrast agent. In another work black phosphorous based QDs (BPQDs) were developed to attach MNPs for PTT/MH applications.^219^ In this work they reported that BPQDs are a suitable choice for laser absorption center for PTT whereas MNPs are also applied for MH therapy. Besides MRI guiding these materials were also showing high precision tumor targeting. Cheng *et al.* devolved MNPs for multimodal imaging where iron oxides are attached to NaYF_4_ and Au thin layers.^220^ For better compatibility and stability these nanoparticles were coated with PEG and used as imaging probes. Moreover, as Au nanoparticles were there, it was also considered for PTT agent.

Zhang *et al.* synthesized manganese doped iron oxide nanoparticles for synergistic application of HT and PTT.^221^ This work reported preparation of MNPs coated with denatured BSA and used for *T*_1_ imaging and PTT. *In vitro* experiment showed that these MNPs were high in MRI capability because of their *r*_1_ value of 8.24 mM^−1^ s^−1^. Importantly, the ratio of *r*_2_/*r*_1_ was calculated to be 2.18, and such a low ratio indicating that MnIO nanoparticles can be applied as *T*_1_ MRI contrast agent. These nanoparticles exhibited brilliant positive *T*_1_ contrast improvement (Fig. 17b) and the brightness of MR images was improved as the concentration of MnIO solutions increased. This dose-dependent color change was because of the relaxation of water proton was enhanced with increasing the dose concentration. *In vivo* photothermal therapy of the MnIO-dBSA composites was examined on a 4T1 tumor bearing mice. The tumor site was irradiated with NIR laser after 20 min of intratumorally injected nanoparticles. Fig. 17c displayed the IR thermal images recorded at the end of 5 min irradiation. In case of MnIO-dBSA treated mice, the surface temperature of the tumor site rose ∼70 °C whereas the temperature of the PBS treated tumor region only reached to 43 °C. After the laser treatment the tumor sites of all the mice turn into black and healed and developed a scab ultimately in a week.

Immunotherapy is an approach to guide immune activation against tumor growth and metastasis *via* mobilizing natural immune cells of the body. Research on cancer immunotherapy has grown in recent years owing to its benefits over traditional treatments including higher efficiency and specificity and decreased toxicity in comparison to radiotherapy/chemotherapy.^222,223^ There are many mechanisms of actions for immunotherapy within tumors applying MH.^224–226^ These methods possess the capability to attain potent immunomodulation without the addition of drugs that cause harmful reactions. Guo *et al.* reported a photothermally activated immunotherapeutic paradigm based on magnetic-responsive immunostimulatory nanoagents (MINPs) integrated with superparamagnetic IONPs and cytosine-phosphate-guanine oligodeoxynucleotides.^227^ In the presence of exterbnal magnetic field, the MINPs exhibited good magnetic targeting ability resulted in high accumulation of the photoabsorber (IONPs) and immunoadjuvant in the tumors for accurate bimodal imaging. Upon NIR exposure, the MINPs assisted photothermal destruction of the primary tumors and releasing tumor related antigens and displaying ‘autologous cancer vaccine’-like functions; therefore, activating antitumor immune response in the presence of cytosine-phosphate-guanine oligodeoxynucleotides containing immunostimulatory nanoagents. In another report Nie and coworkers developed a strategy to improve the treatment of adoptive T cells for solid tumors.^228^ The authors discussed that firstly magnetic nanoclusters were armed with PD-1 antibody (aP) by pH-sensitive benzoic–imine bond and inverse electron-demand Diels–Alder cycloaddition and then bound to the T cells surface through the interaction between aP and PD-1. Due to the magnetic property the T cells, along with aP was magnetically directed to the tumor sites with MRI guiding. Throughout intratumoral infiltration, the acidic extracellular environment caused hydrolysis of the benzoic–imine bond which leads to the discharge of aP. So, the adoptive T cells and aP functioned in a synergistic way, and the growth of solid tumors could be slowed down with minor side effects. Jin *et al.* developed MFNPs (α-AP-fmNPs) loaded with antigen peptide, IONPs and indocyanine green (ICG).^229^ In the context of dendritic cell (DC) based immunotherapy, α-AP-fmNP loaded DCs showed antigen presentation ability, magnetic pull force (MPF) responsiveness, optical and MRI detectability. Under MPF treatment, *in vivo* migration efficacy of α-AP-fmNP-loaded DCs was increased significantly, causing enhanced antitumor efficiency.

## 13. Hyperthermia induced immunotherapy

Hyperthermia based radiotherapy and chemotherapy are quite popular these days adopted by clinicians. Hyperthermia has been used as a medical tool in immunotherapy where their synergy works excellently. Hyperthermia excited a wide range of antineoplastic immune responses throughout the clinical temperature range which are non-harmful to animal skins. Hyperthermia already shows several *in vitro* and *in vivo* cases with excellent cancer immunity responses. But still there is a leading question of how to combine hyperthermia and immunotherapy to promote them in a single diagnostic tool? Recently hyperthermia and immunotherapy are combined used to treat stage I and stage II cancers. In magnetic hyperthermia (MHT) magnetic nanoparticles are playing the crucial role. These MNPs are getting heated in presence of external oscillating magnetic field. This heat energy comes as a result of internal collision among the magnetic domains inside the multidomain nanomagnets. But the main limitation of using the MHT is they can treat tumor growth in primary conditions, not for distant metastatic growth.^230^ Moreover, it has is also lethal to the cancer patients to treat distant metastatic growth.^231^ There are some cutting edge outcomes proposed by the clinicians based on the immunotherapy like cancer vaccines,^232^ chimeric antigen receptor T cell engineering treatment 30, and immune checkpoint blockade (ICB) strategies.^233^ Morita *et al.* revealed at 2001 that MHT assisted ablation could raise the localized concentration of lymphocytes in tumor area which is due to penetration of lymphocytes to targeted area.^234^ This was inferred that thermoablation caused by MHT promotes the release of tumor associated antigens (TAA). TAA are arranged by dendritic cells (DCs) followed by association to T-cells.^235^ Sometimes, T-cells are acting as immune checkpoint blocking agent which improves the immune response.^236^

Several researchers already established that cell death *via* immunogenic fashion is an outcome of hyperthermic thermoablation.^237^ Immunogenic cell death (ICD) is an irreversible process where damage associated molecular patterns (DAMPs) are secreted which transforms a cell from non-immunogenic to immunogenic in nature.^238^ MHT causes formation of some endogenic molecules like denosine triphosphate (ATP), DAMPs, calreticulin (CRT), high mobility group B1 (HMGB1), heat shock protein 90 (HSP90), and HSP70. These molecules have immense importance to hold the cellular integrity in ambient circumstances.^239^ Dong *et al.* reported Ag_2_Te QDs based photonic thermal ablation for 4T1 breast tumor with a tumor suppression rate 94.3%.^240^ Chen *et al.* reported black phosphorus based QDs based targeted thermoablation therapy by MHT.^241^ Here, the QDs were conjugated with gambogic acid and encapsulated with copolymer soft matrix. The effect of thermoablation was observed to start within 2.5 min as reported.

## 14. Magneto-fluorescent nanoparticles as delivery cargoes

Cell tracking, guiding, and external stimuli responsiveness are the recent scientific breakthrough in cancer research. The most common format for these particles is forming a magnetic core and that was decorated by quantum dots/carbon dots. These particles are magnetic as well as fluorescent, biocompatible, easy surface engineered, and non-photobleachable. The drug encapsulation into these particles is carried out either physical/electrostatic interactions or pure covalent grafting. Covalent interaction makes the release of drug molecules from magneto-fluorescent assembly is comparatively better controlled than physical bonding. Ye *et al.* prepared Mn : ZnS QDs and SPIONs assembly coated with poly(lactic-*co*-glycolic acid) copolymer for drug carrier.^242^ They studied the release of busulfan as a model drug molecule. These copolymer magneto-fluorescent vesicles were showing ∼80% drug release in 6 h. The similar kind of QDs were also applied in another work where the authors reported silica coated magnetite MNPs coated with LCST copolymer assembly and studied their doxorubicin release kinetics.^192^ They reported ∼88% encapsulation efficiency and ∼15 wt% loading capability. Li *et al.* reported polyelectrolyte coated CdTe QDs attached magnetite MNPs for pH-sensitive drug release device.^33^ Yang *et al.* developed polycaprolactone (PCL) coated MNPs having fluorescent behavior for anticancer activity. They reported synthesis of CdTe attached iron oxide that was coated with PCL for preparing microcapsules and used for tamoxifen anticancer drugs.^81^ They developed microfluidic devices for production of almost homogenous size distribution of microcapsules. Micellar encapsulation of QDs into MNPs are also prepared by a group of researchers that was applied for doxorubicin delivery.^243^ Huang *et al.* prepared GQDs based targeted nanoparticles for doxorubicin delivery. They attached gadolinium and folic acid to the nanoparticles for guided therapy of cancers.^141^ Magneto-fluorescent MNPs based semi-IPNs were synthesized for pH-sensitive release of anticancer drug temozolomide.^244^ Yao *et al.* synthesized apoferritin based MNPs for doxorubicin delivery. They used gadolium doped QDs as a magneto-fluorescent component and attached to apoferritin for introducing pH-sensitive character of the MNPs.^182^ Fakhri *et al.* reported Ag_2_O QDs decorated MNPs and cellulose nanofibers that was ferromagnetic in nature.^245^ They loaded dual drugs that showed more than 60% drug release within 24 h. Ag_2_O QDs have antioxidant property besides fluorescence that helped the MNPs to be an antioxidant as well as magneto-fluorescent. In another work a biopolymer named chitin was hydrothermally pyrolysed to get carbon dots that was coupled to Gd, Eu, and Mn for magneto-florescent nanoparticles. These particles were tuned to targeted system after attaching folic acid onto its surfaces. Gd-doped QDs showed excellent magneto-fluorescent behavior as well as good drug encapsulation.^246^ The cumulative release profile from these MNPs showed also pH-sensitive release. It had been seen that with increasing the pH, the cumulative release % was minimized; it inferred the MNPs were suitable for physiological environment. Das *et al.* synthesized boronic acid functionalized MNPs based on gadolinium that was excellent drug carrier for 5-fluorouracil (5-FU).^57^ This material was used for pH-sensitive drug delivery. In a brief magneto-fluorescent nanoparticles could be a next generation surface engineered particles for multimodal applicability.

## 15. Magnetolytic therapy: innovative technology in cancer therapeutics

**Fig. 18:**
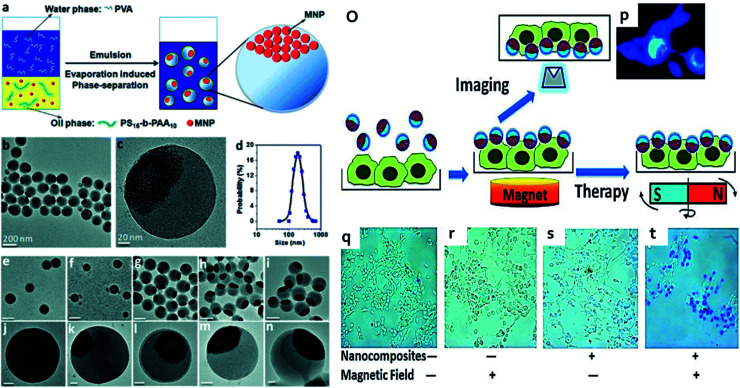
(a) Schematic illustration of the preparation of multifunctional nanocomposites with spatially separated functionalities. (b and c) TEM images of the nanocomposites at different magnifications (d) hydrodynamic size (e–n) with increasing concentrations of MNPs, the size of the nanocomposites increased and their magnetic segments. Scale bars: (e–i) 200 nm and (j–n) 20 nm. (o) Schematic illustration of the experimental conditions and magnetolytic therapy. Nanocomposites are first brought down to cell surface by magnetic attraction. Cells can then be examined by fluorescence microscopy or treated with oscillating magnetic fields, which result in mechanical forces to break down cell membranes. (p) Fluorescence imaging of cells coated with nanocomposites. (q–t) Magnetolytic therapy on tumor cells. Reproduced from ref. 247 with permission from American Chemical Society, copyright 2010.

The rapid progress of synthetic approaches of several therapeutic agents has led advancement of a series of interesting nanomedicine over the past few years. Hu and coworkers first introduced the term ‘magnetolytic therapy’.^247^ This technology involves magnetic field modulated imaging and an innovative prospect to apply this technology in cancer cell therapeutics subjected to magnetically governed mechanical forces. The authors developed a magneto-optical combination by introducing pyrene and MNPs in separate compartments based on microemulsion, surfactant free and controlled phase separation. Fig. 18a demonstrated the synthetic route of the biphasic nanocomposites. TEM images (Fig. 18a and b) of the nanocomposites disclosed the biphasic structure with MNPs accumulated on one side and polymers on the other side. DLS study confirmed that the particles have hydrodynamic diameter of ∼190 ± 38 nm and good colloidal stability in aqueous solution. TEM images (Fig. 18e–n) clearly displayed compositional tailoring of the magneto-optical biphasic particles that increasing concentrations of the MNPs resulted in larger MNP phases and particle sizes. Using biphasic nanocomposites the simultaneous imaging and cancer cell treatment is shown in Fig. 18o–t. The schematic representation of the magnetolytic therapy was depicted in Fig. 18o. At first nanocomposites were attached to the cell surface by magnetic attraction. Cell were then studied by fluorescence microscopy/treated with oscillating magnetic fields that cause mechanical forces to break down cell membranes. Fluorescence imaging of the cells (Fig. 18p) confirmed that nanocomposites can coat the cell surface. To analyse the therapeutic effect of the particles, cells were placed in a spinning magnetic field (50 rpm) that generates a mechanical force on the cell membrane. After 15 min exposure to the spinning magnetic field, the majority of the tumor cells were killed. The dead cells were identified by trypan blue staining (Fig. 18t). In comparison with the control experiments, missing either the nanocomposites or magnetic fields no significant effect on cell viability was observed (Fig. 18q–s). Later, Zhao and coworkers developed one dimensional degradable dextran-Fe_3_O_4_ nanohybrids for magnetolytic therapy.^248^ In comparison to the spherical Fe_3_O_4_@Dex-PGEA as the counterpart, 1D FDP illustrated good cellular uptake efficacy, which can also rotate under an alternating magnetic field to produce a shear force to terminate tumor cells and show good photothermal features. Future improvement of these kind of newfangled multifunctional nanocomposites contains targeted imaging and therapy *in vitro* and *in vivo* and we envision that this technology will expose prospects in nanomedicine, catalysis. Spintronics, electronics, and multimodal imaging.

## 16. Summary and future perspective

The inspiration of this review comes from the numerous cutting edge research carried out throughout the whole world. The discussion could provide a clear idea of the synthesis of fluorescent nanoparticles and how to impart magnetism into them. The initial study in this review covers up the various synthetic and morphologies of the MFNPs and MFQDs and their magneto-optical behaviors of physiologically simulated conditions. MFQDs are the younger members of the fluorescent nanomaterials family. The synthesis of MFQDs is based on two approaches; one is based on inorganic precursors whereas another one is purely carbon based. The optical properties especially the FL character of the QDs were tuned after attaching surface active ligands. Surface active ligands were ionic liquids, polymers (anionic/cationic/neutral), proteins, antibodies, and small molecules. According to the synthesis procedures, they were also categorized into *in situ* and *ex situ* techniques. In case of *in situ* nanoparticles having QDs' precursors and magnetic components were solvothermally treated to prepared magneto-florescent nanoparticles. But for *ex situ* technique, the nanoparticles were prepared in two consecutive steps; initially they were prepared followed by some surface decoration of ligand modification. These MFQDs are biocompatible that made them easy acceptability towards biological or *in vivo* works. MFQDs are easily permeable/attach to cancer cells which actually enhance the effect of bio-marking of the tumors. The magnetism of these particles was manipulated by adjusting their size and shape which is directly related to their transition from ferromagnetism to superparamagnetism. Superparamagnetism is always welcomed due to its transient moment deepening upon the external field. Thus, superparamagnetic iron oxide nanoparticles (SPIONs) are a good replacement of commercially used Gd based MR agents. MFQDs are also drawing interests for the clinicians because of their efficacy in fundamental biomedical researches especially in sophisticated imaging techniques. Herein, the bioimaging platform also has been nurtured in light of their *in vivo* imaging qualities. The magnetic fluorescent nanomaterials are also surface engineered resulting in bifunctional nanoparticles that are the most highlighted in multimodal theragnostic probes. Bifunctional nanoparticles are categorized as heterocrystals and Janus particles. The beauty of these particles is in their surface functionalities that could anchor more than one ligand in same nanoparticles. These have an immense applicability not only in theragnostic but also in the cancer research. Inorganic heterocrystals and Janus particles are still under research for obtaining the better futuristic technologies.

Besides the theragnostic approaches, MFNPs and MFQDs are also exploited in hyperthermia and magnetolytic technologies. In this review we also discussed the classical approaches of hyperthermia and how MFQDs gear up the research into non-invasive apoptotic strategy for carver patients. Hyperthermia is a non-contact heating mode that could heat magnetic nanoparticles penetrated cells to death in a precise manner. Magnetolytic therapy is a magnetic field guided imaging technique exclusively applied for cancer research. It is actually quite effective for fluorescent intensity modulations and altering orientations without affecting system morphology and intrinsic features. Combining of magnetic and optical properties could impose several biomedical marvels and that's why ‘single particle-multimodality’ would become a daunting challenge for all materials scientists.

Although numerous nanostructures and applications of magnetic nanolights have been developed, further study is still necessary for their upconverting optical properties and versatile design strategies. (i) The decrease of quantum yield of the fluorescent particles was attributed to the surface quenching effect, which is important for nanosized QDs due to their large surface-to-volume ratio. Simple way to improve the quantum efficiency of nanosized particles and more strategies are required with an optimized architecture. (ii) Advancement of the existing fabrication approaches to enable large scale, controllable sized magnetic nanolights. (iii) Investigation into the prospective characteristics to enable the transformation between laboratory research and practical applications. (iv) Novel strategies to fabricate Janus particles having magnetic and fluorescent characteristics are required with an optimized manner to increase their application in diverse fields. (v) Recently the deep penetration depth of NIR light has been illustrated, it is noteworthy that the interaction between light and tissues is rather complex. In this context, dual emission ratiometric nanoprobe can minimize the errors originating from different wavelengths and may deliver an approach to acquire accurate signals especially for the *in vivo* applications. (vi) The conjugation of different components for the most bifunctional MFNPs generally needs soft or hard linker, that includes complex preprocessing on each component. Additionally, for the cases where linker is not required, the fabrication of bifunctional MFNPs still demands troublesome crystal growth process. Therefore, facile and proper emerging methods for the synthesis of MFNPs with high efficacy will be of great importance. (iv) Concern about the biosafety is one of the main obstacles that bound the clinical use of these MFNPs, although they are widely used in biomedical applications. The integration of the MFNPs in the physiological environment still raises probable biocompatibility issues. To obtain ideal therapy efficiency, the high-power density of excitation light and high dose of nanostructures are essential which restricts their application in clinical use. Thus, it is essential to advance highly capable magnetic nano lights that can be employed under low irradiation fluence to enlarge the biological applications. (v) Although MFNPs possess good physicochemical properties but their therapeutic applications are restricted because of toxicity, cost, and nonbiodegradability. To address these issues, self-fluorescent polymers are good substitute to the toxic organic dyes and quantum dots to achieve MF nanocarriers. Wang and coworkers developed MF nanocarriers based on conjugated self-fluorescent polymer poly(fluorene-benzothiadiazole) and IONPs for multimodal tumor imaging.^249^ Another strategy to increase the biocompatibility is PEGylation of IONPs. Vijayan *et al.* used self-fluorescent PEGylated aliphatic polymer to impart both stability as well as fluorescence property to IONPs.^250^ This technique avoids the usage of toxic heavy metal QDs or commercial organic dyes that are requisite for the construction of MFNPs and endowed for potential theranostic applications.

The lanthanide ion Gd^3+^ has high magnetic moment, symmetric electronic ground state and therefore they are extensively used for application like bioimaging and MRI contrast agent. Gd^3+^ is toxic and therefore to decrease the toxicity and increase stability surface-chelated Gd^3+^ ions could be complexed in organic chelates that coordinate to the paramagnetic ions. Another strategy to attain bimodality is by attaching paramagnetic chelates to QDs both covalently and non-covalently. Also, Gd-doped CQDs could be another alternative to reduce Gd leakage even under complex biological environments by virtue of the inertness of carbon cages. It is known that longitudinal relaxivity mostly influenced on the direct interactions between Gd^3+^ and hydrogen protons proposing that only outer surface-chelated Gd^3+^ could contribute to longitudinal relaxivity. Thus, by reducing the total amount of Gd^3+^ in Gd-doped unit while increasing their surface-chelated Gd^3+^ would enhance their practical applications.

Recently, different kinds of luminescent materials such semiconductor QDs, organic fluorophores have been coupled with magnetic units to construct MFNPs that can function both for magnetic and optical probes. In many cases, these QDs contain heavy metals such as Cd, Pb *etc.* involves potential toxicity, less water solubility and environmental hazards that are hindering their applications. In this context, CQDs, heavy metal free carbon nanomaterial can be a superior framework over conventional semiconductor quantum dots to construct multi-modal imaging probes, due to their advantages excellent biocompatibility, bright fluorescence, simple synthesis methods and ease of surface functionalization.

## Conflicts of interest

There are no conflicts to declare.

## Supplementary Material
